# Image analysis optimization for nanowire-based optical detection of molecules

**DOI:** 10.1515/nanoph-2024-0243

**Published:** 2024-09-30

**Authors:** Rubina Davtyan, Nicklas Anttu, Julia Valderas-Gutiérrez, Fredrik Höök, Heiner Linke

**Affiliations:** 5193 Division of Solid State Physics, Lund University, P.O. Box 118, SE-22100 Lund, Sweden; Physics, Faculty of Science and Engineering, Åbo Akademi University, Henrikinkatu 2, FI-20500 Turku, Finland; Department of Physics, Chalmers University of Technology, SE-41296 Göteborg, Sweden; NanoLund, Lund University, P.O. Box 118, SE-22100 Lund, Sweden

**Keywords:** nanowire, fluorescence microscopy, epifluorescence, TIRF, biosensing, image analysis

## Abstract

Semiconductor nanowires can enhance the signal of fluorescent molecules, thus significantly improving the limits of fluorescence detection in optical biosensing. In this work, we explore how the sensitivity can further be enhanced through “digital” detection of adequately spaced vertically aligned nanowires, employing single-emitter localization methods, and bright-field microscopy. Additionally, we introduce a systematic analysis pipeline aimed at harnessing this digital detection capability and evaluate its impact on detection sensitivity. Using a streptavidin-biotin assay, we demonstrate that single-emitter localization expands the dynamic range to encompass five orders of magnitude, enabling detections of concentrations ranging from 10 fM to 10 nM. This represents two to three orders of magnitude improvement in detection compared to methods that do not utilize single-emitter localization. We validate our analysis framework by simulating an artificial dataset based on numerical solutions of Maxwell’s equations. Furthermore, we benchmark our results against total internal reflection fluorescence microscopy and find, in time-resolved titration experiments, that nanowires offer higher sensitivity at the lowest concentrations, attributed to a combination of higher protein capture rate and higher intensity per single protein binding event. These findings suggest promising applications of nanowires in both endpoint and time-resolved biosensing.

## 1 Introduction

Fluorescence microscopy is widely used in optical biosensing [1], [2], [3], with single-molecule biosensors being of high significance due to their ability to detect and characterize extremely low concentrations of analytes [4], [5]. Nanostructured surfaces can improve the limit of detection (LoD) of conventional fluorescence microscopy techniques by amplifying the fluorescent signal intensity and by increasing the surface area available for the interaction [3], [6], [7]. This paper is concerned with the specific example of semiconductor waveguiding nanowires [8], [9]. Vertical nanowires with a high refractive index, and with a diameter matched to the fluorescence wavelength, have been found to enable an order-of-magnitude increase in fluorescence intensity compared to flat surfaces, and are able to detect single-molecule binding events in widefield fluorescence imaging [8], [9], [10]. The physical mechanisms underlying this signal amplification by semiconductor nanowires are now well understood [11], and the following contributing factors have been identified: (i) the ability of nanowires to act as waveguides and to emit light directionally [12], [13], (ii) excitation enhancement [14], [15], and (iii) emission modification by quantum yield enhancement [16].

**Figure 1:**
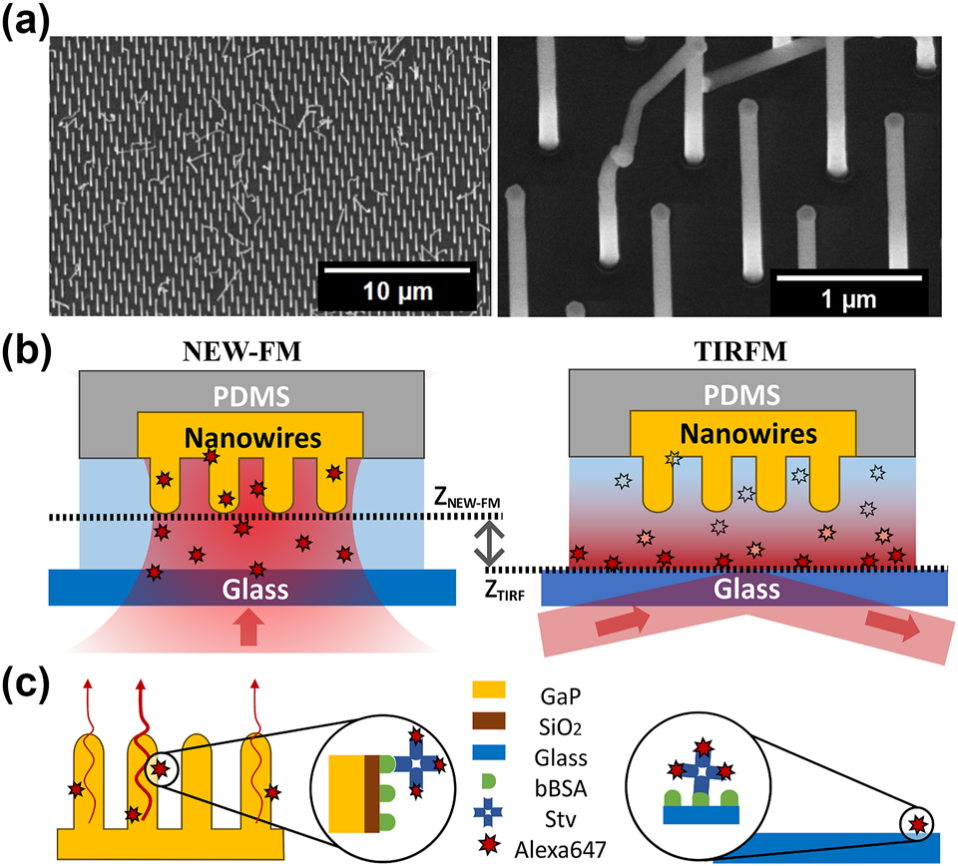
Experimental setup. (a) SEM images of GaP nanowires at different magnifications. The distance between nanowires is around 1 µm, which ensures lateral optical resolution. Around 85 % of the nanowires are vertically aligned and equally spaced, but the remaining 15 % are kinked or missing, thus do not contribute to signal enhancement. (b) Illustrations of the two imaging modes used to analyze molecular binding in a microfluidic channel. NEW-FM refers to nanowire-enhanced widefield fluorescence microscopy, where the focal plane of the objective is aligned with the tips of nanowires. For TIRFM, the focal plane is aligned with the glass surface, and an evanescent field is created at the glass–water interface. Two images (one with NEW-FM on a nanowire surface and one with TIRFM on glass) were acquired every 45 s in the same microfluidic channel. (c) Schematic of the surface functionalization: biotin-conjugated bovine serum albumin (bBSA) is adsorbed to the SiO_2_ coating on nanowires and on microscope coverglass, covering both the nanowires and the substrate. The analyte, which is a streptavidin labeled with the dye AlexaFluor647 (StvA647), is bound to bBSA with high affinity.

However, in addition to these physical enhancement effects, one can also expect possibilities to improve the LoD based on the “digitalization” of the signal [17], [18], [19], [20] arising from a large number of individual vertically aligned nanowires with lateral separation larger than the diffraction limit of light (Figure 1(a)). In this study, we aim to understand and quantify the advantages of signal digitalization due to many individual nanowires by using single-emitter detection techniques [21]–[25] combined with image pre- and postprocessing using nanowire position information [26], [27]. We validate the performance of our image analysis pipeline using simulations of nanowire-bound fluorophores and demonstrate a large dynamic detection range, where the application of single-emitter methods seems to be the most valuable at low concentrations below 10 pM.

To validate the performance of nanowire-enhanced widefield fluorescence microscopy (NEW-FM) further, we benchmark our approach against total internal reflection fluorescence microscopy (TIRFM) [28] in time-resolved binding experiments. To enable direct comparison, we perform NEW-FM and TIRFM (Figure 1) simultaneously, in the same microfluidic channel and in the same experimental conditions (Figure 1(b) and (c)). We find that NEW-FM provides twofold higher sensitivity compared with TIRFM.

## 2 Benefits of digital image analysis for nanowire images

To quantify the fluorescent signal in each image, we conducted detection and localization of nanowires that have one or more fluorophores bound to them (labeled analyte molecules in Figure 1(c)). In principle, and in practice, as we will demonstrate below, three regimes can be distinguished. At very low analyte concentrations (Regime I), the signal originating from an individual nanowire represents a single molecule bound to the nanowire [8], whereas at high concentrations, the signal comprises the collective emission from multiple bound molecules (Regime III). Intermediate conditions emerge (Regime II) when most of the nanowires already have one bound molecule, thereby increasing the probability that some nanowires have multiple bound molecules.

In Regime I, the signal enhancement of an individual nanowire compared to a free fluorophore can be described by the combination of optical enhancement effects denoted by *σ*_*enh*_ [11]. In Regime III, when multiple fluorophores are bound to a single nanowire, the resulting image is the sum of the intensities of individual fluorophores, and the signal from each fluorophore is enhanced individually. In Regime I, we can utilize the digital counts of nanowires as a measure of signal intensity, whereas in Regime III, the intensity of individual nanowires is most suitable. Therefore, we choose the following signal quantification metrics: (i) the digital count of bright nanowires (*N*), (ii) the average intensity per bright nanowire $\left({\bar{I}}_{i}\right)$ characterizing the number of bound fluorophores, and (iii) the overall intensity emitted from bright nanowires (*I*_tot_).

**Figure 2:**
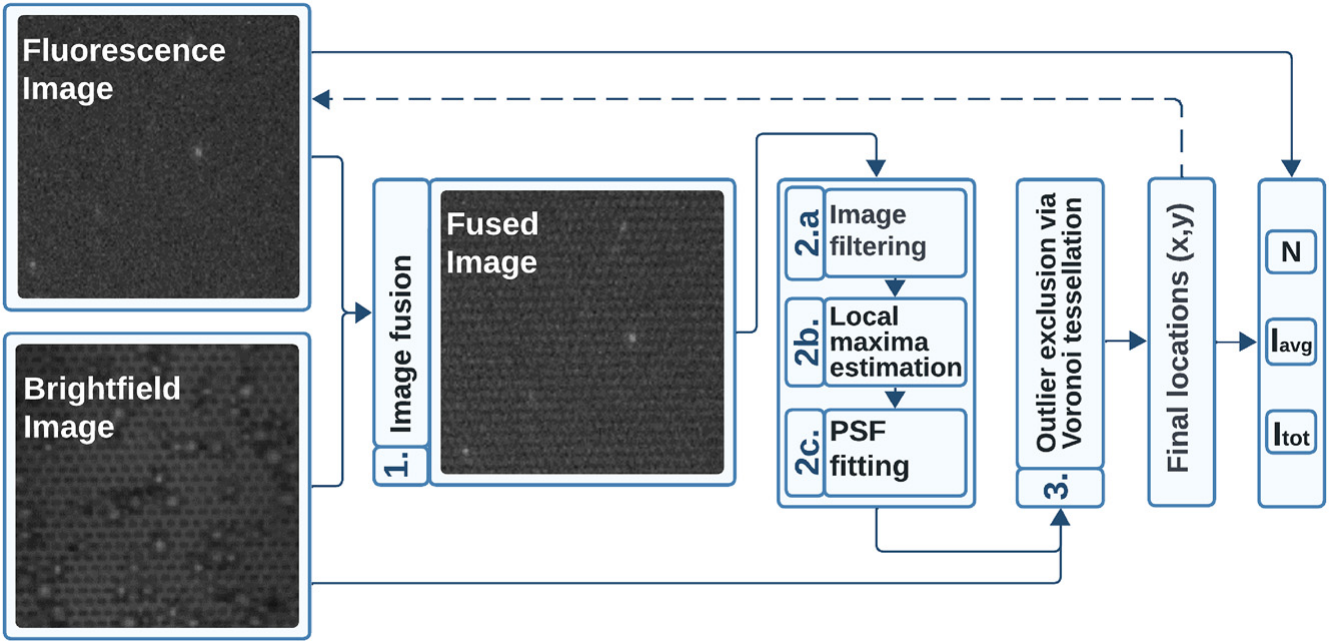
Pipeline for single nanowire localization. Bright-field image is used in and optional preprocessing step to enhance the signal originating from nanowires (step 1), followed by single-emitter localization (step 2) and outlier exclusion via Voronoi tessellation (step 3). See main text, as well as Supplementary Methods, Sections 1.3, 2, and 3.1, for a more detailed description of the analysis pipeline).

The determination of *N*, ${\bar{I}}_{i}$ , and *I*_tot_ requires precise single-emitter localization techniques [21], [22], [29]. The overall analysis pipeline consists of the following steps (see Figure 2):

1. **Image fusion:** The image is preprocessed with Fourier domain techniques [26], [30] to selectively enhance the signal originating from nanowires
2. **Single-emitter localization pipeline**, which consists of the following commonly used techniques [21], [22]:
  (a) Image filtering, using à-trous wavelet filtering [31],
  (b) Local maxima estimation, using local gradient thresholding [23],
  (c) Point-spread function (PSF) fitting, via Gaussian Maximum Likelihood Estimation [24], [25].
3. **Outlier exclusion** via Voronoi tessellation [32] to suppress the signal that is not colocalized with the nanowire tips.

Steps 1 and 3 rely on the availability of bright-field images and the periodicity of nanowire placement and are optional (see Supplementary Methods, Section 1.4 https://github.com/nanoRuby/NanoLoci). The full analysis pipeline in Figure 2 is referred to as *NanoLoci* and is also provided as a MATLAB software package with a graphical user interface.

## 3 Methods

### 3.1 Signal quantification metrics

Once the locations of fluorescent nanowires are obtained, the signal can be evaluated. Kinked or missing nanowires, which fail to enhance the signal due to disrupted geometry, appear as undetected dark spots in image analysis, thus are normally below the detection threshold and remain unaccounted for in the signal quantification. To quantify the signal of individual bright nanowires, we calculate the average pixel intensity ${\bar{I}}_{i}$ for each successfully localized nanowire:

(1) $$\forall i\in N,\;{\bar{I}}_{i}=\frac{\overset{K}{\underset{k=1}{\sum }}\left(I_{i,k}-I_{DC}\right)}{K}$$

where *I*_*i*,*k*_ represents the individual pixel intensity of each *k*th pixel in the local neighborhood *K* of *i*th nanowire, and *N* is the total number of bright nanowires. In our analysis, we used a window of size 3 × 3 pixels around the nanowire location, thus *K* = 9. In Regime II and III, ${\bar{I}}_{i}$ increases alongside *N* and is expected to be proportional to the number of bound molecules to each individual nanowire:

$${\bar{I}}_{i}\approx <\sigma_{\mathrm{enh}}>\cdot \overset{M}{\underset{j=1}{\sum }}I_{j}^{fl}$$

Here, $<\sigma_{\mathrm{enh}}>$ is the combined enhancement factor due to all optical enhancement effects and represents *σ*_*enh*_ averaged over the axial positions of fluorophores [11]. $I_{j}^{fl}$ is the intensity of an individual fluorescent molecule, and *M* is the expected number of molecules bound to each nanowire.

As *N* and ${\bar{I}}_{i}$ capture two different quantities sensitive to analyte concentrations in two different regimes, we also introduce the overall fluorescence signal *I*_tot_, which incorporates nanowire digital count and intensity integration along the nanowire axis:

(2) $$I_{\text{tot}}=\overset{N}{\underset{i=1}{\sum }}{\bar{I}}_{i}$$

We also use $I_{\text{avg}}=\frac{I_{\text{tot}}}{N}$ to quantify the average intensity per individual bright nanowire across the whole specimen.

In order to obtain the relative signal from the specimen compared to the blank, we acquire and analyze images before adding the fluorescent analyte and determine the number (*N*_0_) and the total intensity *I*_0,tot_ for each blank sample. The normalized values for each specimen are then:

(3) $$\begin{array}{ll} N^{\prime } & =N-N_{0}\\ I_{\text{tot}}^{\prime } & =I_{\text{tot}}-I_{0,\text{tot}} \end{array}$$

In time-resolved processes, we omit image pre- and postprocessing steps (steps 1 and 3 in Figure 2) for simplicity and direct comparison with TIRFM, as image fusion and outlier exclusion algorithms are not applicable for glass surfaces. See Supplementary Methods, Section 1.6 for additional information on data analysis.

### 3.2 Nanowire growth and characterization

Gallium phosphide (GaP) nanowire were grown using metalorganic vapor-phase epitaxy (MOVPE) from gold (Au) seed nanoparticles of density 1.19 Au seeds/µm^2^ on 3″ (111) GaP substrates in an Aixtron 200/4 reactor (Aixtron, Herzogenrath, Germany) [33]. GaP nanowires and the substrate from which they are grown were coated with a SiO_2_ layer of thickness *d*_coat_ = 10 nm using atomic layer deposition (ALD, Fiji, Cambridge Nanotech, Cambridge, MA, USA) to provide an adsorptive surface for molecular binding. Values for the nanowire diameters at the top (*d*_top_) and bottom (*d*_bot_) and other dimensions are provided in Table 1.

**Table 1:** Morphological characterization of nanowire platforms using scanning electron microscopy (SEM) images.

Morphological characterization of nanowire platform	
Diameter on the top, *d*_top_, [nm]	114.41 ± 2.22
Diameter on the bottom, *d*_bot_, [nm]	122.41 ± 2.30
Coating thickness, *d*_coat_, [nm]	10.48 ± 1.11
Length, *L*, [µm]	2.50 ± 0.06
Density of all nanowires, *ρ*, [*#*/µm^2^]	1.19 ± 0.01
Density of straight nanowires, *ρ*_*S*_, [*#*/µm^2^]	0.99 ± 0.02
Nanowire spacing, *p*, [µm]	0.99 ± 0.00

The tapering factor of nanowires, calculated as (*d*_bot_ − *d*_top_)/(*d*_bot_ + *d*_top_), yields 0.03, which means that it does not have a negative effect on the signal enhancement properties of nanowires [8].

### 3.3 Surface functionalization and image acquisition

GaP nanowire platforms of size 2.5 × 2.5 × 0.3 mm^3^ were first functionalized with 6 µM biotinylated bovine serum albumin (bBSA) dissolved in phosphate-buffered saline (PBS) for 1 h at room temperature. After incubation with bBSA, the samples were washed with PBS, followed by incubation with the analyte, AlexaFluor647 labeled streptavidin (StvA647, ThermoFisher Scientific, USA), and prepared from 1 mg/mL stock solution. See Supplementary Methods, Section 1.1 for additional details.

#### 3.3.1 Single-frame titration measurements

**Figure 3:**
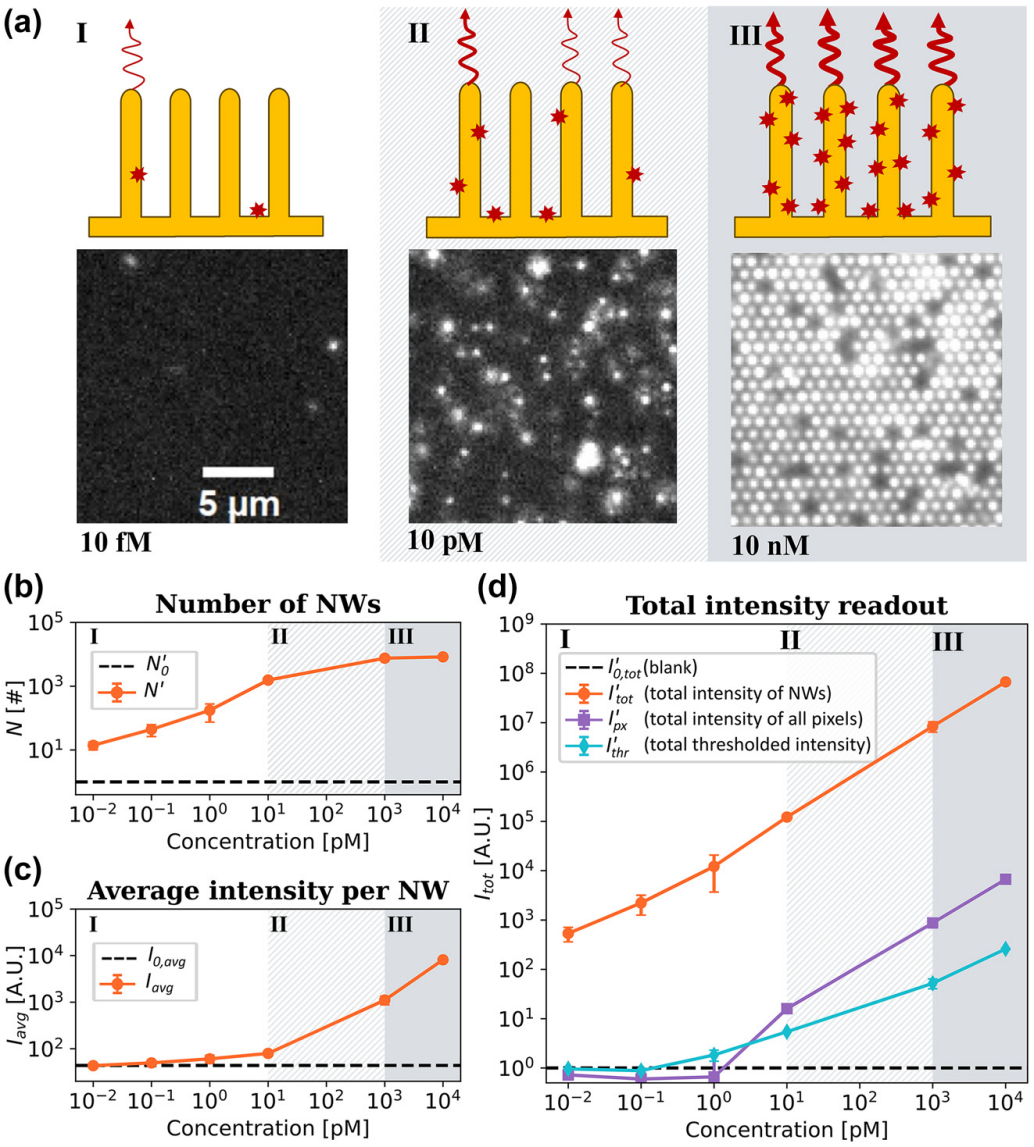
Titration experiments of Stv-Alexa647 binding to a nanowire array. (a) Micrographs representing fluorescence emission upon additions of StvA647 at 10 fM, 10 pM, and 10 nM. The illustrations depict the three regimes identified (see main text). (b) The normalized number of localized bright nanowires (*N*′) versus StvA647 concentration. (c) *I*_avg_, the average intensity per bright nanowire versus StvA647 (orange curve). The dashed black line represents the average intensity of the blank measurements. (d) The normalized total intensity detected from nanowires $\left(I_{\text{tot}}^{\prime }\right)$ , serving as a readout signal (orange curve). Nonsingle emitter methods, such as averaging the pixel values ( $I_{\text{px}}^{\prime }$ , purple) and thresholding averaging the pixel values ( $I_{\text{thr}}^{\prime }$ , cyan), are plotted for a comparison. Detections from blank images were used to normalize the number of molecules (b) and total intensities (d). See Eq. (3) and Supplementary Methods, Section 1.6.1 for additional details on the calculation of intensity metrics and normalization.

The samples were imaged with an inverted epifluorescence microscope (Eclipse Ti2, Nikon), water immersion objective (magnification of 60×, numerical aperture (NA) = 1.2), and a Sona sCMOS camera (Andor, Oxford instruments). A 640 nm laser was used as an excitation source at approximately 10 mW excitation power with 100 ms exposure time. Reference blank images were taken following bBSA incubation and washing step for each individual specimen. Prior to imaging, bBSA-coated samples were prebleached at a maximum laser intensity for 10 s to suppress the nonspecific signal. Seven consecutive concentrations (0.01 pM, 0.1 pM, 1 pM, 10 pM, 1 nM, 10 nM, and 100 nM) of StvA647 were prepared and imaged both in bright-field and fluorescence regimes. For each concentration as well as blank measurements, 6 different regions of size 92.85 µm × 92.85 µm were imaged. The reference images were analyzed and used to normalize the number of detections (*N*) and total intensities (*I*_tot_) in Figure 3(b) and (c) according to Eq. (3).

#### 3.3.2 Time-resolved measurements

The measurements were performed under the same imaging conditions as described above, with the only difference of using an oil immersion objective (magnification of 60×, NA = 1.49). To directly compare time-resolved NEW-FM and TIRFM imaging, two consecutive images of the sample were taken without a time delay, alternating the focal plane between nanowire platforms and glass coverslip. To image the sample over the course of a long time, these images were taken every 45 s. And 1 nM StvA647 was added to the channel and imaged over the course of 40 min. Afterward, a higher concentration of analyte (10 nM) was added. The same location both on nanowires and on glass was imaged during the whole duration of the measurement.

### 3.4 Simulated dataset

In order to evaluate the performance of NanoLoci and other image analysis approaches, we simulated the image formation of fluorophores bound to vertically aligned SiO_2_-coated GaP (*n*_GaP_ = 3.34 at the modeled wavelength of *λ* = 640 nm) nanowires immersed in aqueous solution ( $n_{H_{2}O}=1.33$ ) (see [11] for technical details and summary of the modeling). This has the advantage that simulated images can be used as ground truth, and to unambiguously determine the precision of imaging and image analysis.

The modeling was performed by solving Maxwell’s equations for Alexa647 fluorophore, described as an isotopic dipole in the vicinity of nanowire scatterer using finite-element method (FEM) in COMSOL Multiphysics [11], [14]. The simulations were performed for widefield microscopy, modeling the incident light as a combination of incoherent plane waves within the NA of the objective. We modeled GaP nanowires of diameter *d*_top_ = *d*_bot_ = 100 nm, as the tapering of the nanowires is negligible in terms of the waveguiding of nanowires [8]. The length of the nanowires was set at *L* = 3 µm, and the thickness of the coating layer was *d*_coat_ = 10 nm. Given the lateral separation of the nanowires (*p* = 0.99 µm), no interference effects were considered between the individual nanowires.

The excitation enhancement was obtained from modeling the electric field $|\vec{E}{|}^{2}$ at the fluorophore position, separately for each incident angle allowed by the numerical aperture of the objective and two polarization states similar to Refs. [14], [34]. The emission modeling was done for fluorophores bound to different axial positions on the nanowire, with 50-nm stepsize. A near-to-far-field transform is performed using the RETOP package [35]. The Purcell factor (C_Purcell_), representing the modification of emission compared to a reference medium (H_2_O in this case), was calculated and used to obtain the emission enhancement based on the modification of the quantum yield of the fluorophore.

The optical image is created from far-field solutions of Maxwell’s equations [36], [37] in the Fourier space. This leads to an image of nanowire-modified fluorescence for each given axial position proportional to the intensity of the electric field at that location for a given objective (water immersion, NA = 1.2).

Photons were sampled from the modeled image (numerical PSF) onto discrete imaging pixels corresponding to the imaging camera’s pixel size using the generation of random numbers from discrete 2D functions [38]. The excitation and quantum yield enhancement factors, calculated for each *z* position, were used to modify the effective photon count. Different fluorophore concentrations ranging from single molecule binding to multiple binding on a single nanowire were simulated. Each simulated molecule was randomly assigned to either bind to the substrate or to a nanowire. Additionally, the selection of binding sites on individual nanowires, as well as their respective z-coordinates, was determined randomly (see Supplementary Methods, Sections 1.4 and 3.2 for additional details.)

In the event of multiple binding, the photons were sampled from each position and added to form the final image. In case of fluorophore binding to GaP substrate instead of nanowires, we used a conventional *z*-dependent Gaussian PSF [39], [40], as the fluorophores bound to substrate do not couple to nanowire light-guiding modes and no significant enhancement is assumed. Based on the nanowire distribution observed in SEM measurements, 15 % of the nanowires in the simulations were modeled as missing or kinked at random heights. To approximate this, the same *z*-dependent Gaussian PSF used for substrate-bound molecules [39], [40] was applied to the corresponding *z* positions.

The final image (*I*_fin_) is then created by adding the emission from nanowires (*I*_NW_), GaP substrate (*I*_sub_), and imaging noise, including dark current and photon shot noise (see Figure S3 for more details).

### 3.5 Ground truth evaluation metric

We choose Jaccard index (JI), Precision, and Recall as a way to evaluate the performance of the algorithms by comparing the ground truth (GT) to the obtained detections [41]:

(4) $$\begin{array}{cc} \text{JI} & =\frac{\text{TP}}{\text{TP}+\text{FP}+\text{FN}}\\ \text{Precision} & =\frac{\text{TP}}{\text{TP}+\text{FP}}\\ \text{Recall} & =\frac{\text{TP}}{\text{TP}+\text{FN}} \end{array}$$

where TP stands for true positives, which is the number of detections which were matched between the ground truth (locations of waveguiding nanowires) and detected locations. Respectively, false positives (FP) correspond to detections in simulated images, which do not match with ground truth. False negatives (FN) represent the number of nanowire-bound fluorophores present in ground truth but undetected with evaluated algorithms. To obtain the number of TPs, FPs, and FNs in each frame, we calculate pairwise distances between each entry in the ground truth and each detection: $D\left(i,j\right)=\sqrt{{\left(x_{i}-{\tilde{x}}_{j}\right)}^{2}+{\left(y_{i}-{\tilde{y}}_{j}\right)}^{2}};\;\forall i\in M,\forall j\in N$ . Here, (*x*_*i*_, *y*_*i*_) are the locations in ground truth and $\left({\tilde{x}}_{j},{\tilde{y}}_{j}\right)$ are the detected locations. The closest pairs of detections were found based on nearest-neighbors algorithm, if the Euclidean distance *D*(*i*, *j*) was smaller than the threshold (*d*_*M*_). Note, that in this comparison, we only compare the detected locations to true locations without any consideration of intensity values.

## 4 Results

### 4.1 Digital counting and signal integration enables large dynamic range

We performed titration experiments using streptavidin-biotin assay and applied the image analysis pipeline illustrated in Figure 2. *N* and *I*_tot_ were normalized using the corresponding blank measurements according to Eq. (3). For the lowest concentrations (0.01 fM − 1 pM), where less than 10 % of the nanowires are bright, *N*′ increases with increasing concentration (Figure 3(b)), while *I*_avg_ remains essentially unchanged and close to the background level (Figure 3(c)). These concentrations correspond to Regime I, attributed to binding of single molecules to each individual nanowire (Figure 3(a)). Between 1 nM and 10 nM analyte concentration, *N*′ saturates as all nanowires become bright (Figure 3(a) and (b)), while the increase in *I*_avg_ is due to the increase of the number of molecules bound to individual nanowires (Figure 3(a) and (c), Regime III). At intermediate concentrations (10 pM, Regime II), both *N* and *I*_avg_ increase (Figure 3(b) and (c)) as a transition is happening from low to high concentration coverage, resulting both in increasing nanowire counts and average intensity (Figure 3(b) and (c)).

Using NanoLoci, $I_{\text{tot}}^{\prime }$ (see Eqs. (2) and (3)) exhibits a linear response well above background across the full range of measured concentrations, as depicted by the orange curve in Figure 3(d). This linear response spans five orders of magnitude in intensity and analyte concentration, from 10 fM to 10 nM. The comparison between the performance of NanoLoci and methods that do not utilize single wire localization steps, such as pixel averaging or simple thresholding, is illustrated in Figure 3(d). When using average pixel intensity as a signal readout (purple curve in Figure 3(d)), detections are observed from 10 pM, with a dynamic range of two magnitudes extending up to 10 nM. Similarly, intensity thresholding (cyan curve in Figure 3(d)) detects concentrations ranging from 1 to 10 pM to 10 nM, also with a dynamic range of two orders of magnitude, which is 10–100 times less than when single nanowire analysis is performed. Notably, methods not utilizing single emitter localization demonstrate detection above background only starting from Regime II, where most nanowires become bright (Figure 3(c)), and where *I*_avg_ begins to increase (Figure 3(d)).

Thus, we can conclude that the use of single-nanowire localization steps, that is, making use of the digital nature of our images, increases the dynamic range and LoD by two to three orders of magnitude in sample concentration.

### 4.2 Imaging simulations confirm the accuracy of nanowire localization

**Figure 4:**
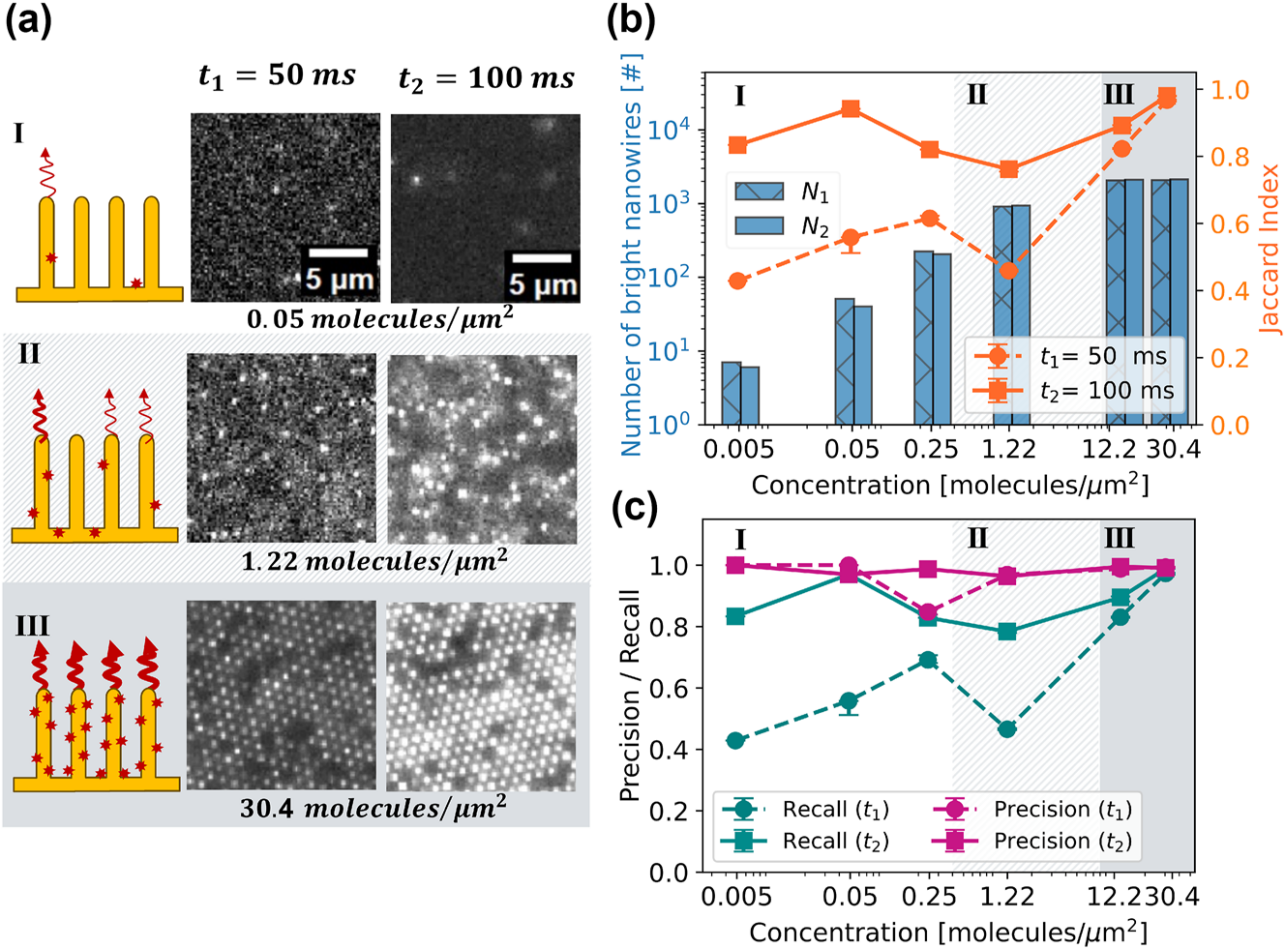
Image analysis verification using simulated dataset. (a) Micrographs representing simulated fluorescence images at surface concentrations of 0.05, 1.22, and 30.4 molecules/µm^2^, with exposure times of *t*_1_ = 50 ms and *t*_2_ = 100 ms. The illustrations represent the three regimes simulated (see main text). (b) The number of bright nanowires, which serves as ground truth (blue, left axis) and JI (orange, right axis) measuring the accuracy of matching detections between the obtained localizations and the ground truth as defined in Eq. (4). (c) Precision and recall curves as defined in Eq. (4) quantify the relation between occurrence of FPs and FNs, respectively.

To verify the performance of NanoLoci (Figure 2, steps 1–3), we simulated a dataset, where the positions of bright nanowires are known. We utilized Jaccard index (JI), Precision, and Recall (see Eq. (4)) as our comparison metrics. Two different exposure times and six analyte concentrations spanning the described three nanowire coverage regimes were simulated. In Regime I, the number of bright nanowires is less than 15 %, and one molecule bound to an individual bright nanowire. In Regime II, where around half of the nanowires are bright, the signal per individual bright nanowire can still vary due to variation of the number of bound molecules. In Regime III, all vertically aligned nanowires are bright due to multiple bound molecules. Nanowires appearing dark in Regime III are clear growth defects unable to enhance the fluorescence of bound fluorophores, thus only contribute to image background (see Figure 4(a)).

We exclusively present the final results of the comprehensive image analysis pipeline, NanoLoci (Figure 2, steps 1–3) photon number. Further insights into the enhanced detection capabilities facilitated by image pre- and postprocessing are available in Supplementary Methods, Figures S8 and 9.

For *t*_2_ = 100 ms, JI is in the range of 0.8–1 across all concentrations (Figure 4(b), solid line). High Precision rates are observed across all concentrations, indicating accurate positive predictions and a low number of FPs (Figure 4(d)). Less than 2 % of all detections are FPs across all concentrations and intensity conditions (see Figure S9(b)). However, Recall decreases at intermediate concentrations (Regime II, Figure 4(c)), indicating that part of the signal remains undetected (see Figure S9(c)). In this concentration regime (Regime II, *c* = 1.22 molecules/µm^2^), around half of the nanowires have one or more molecule (see Table S1).

When the simulated exposure time, and thus the number of emitted photons, is halved, the performance efficiency decreases as well (see Figure 4(b) and (c), dashed lines). The Recall and JI remain high for the highest concentrations (Regime III), where more than 7–15 fluorophores are bound to an individual nanowire. However, both drop by a factor of two in Regimes I and II, which can be attributed to lower number of emitted photons.

The application of the full NanoLoci pipeline (Figure 2, steps 1–3) consistently increases JI by 0.05 − 0.30 (5 − 30 %) in Regimes I and II (Figures S8 and 9(a) and (d)). It is achieved by decreasing the number of FPs and FNs while increasing the number of TPs. However, a number of undetected and falsely detected nanowires still remain (see Supplementary Methods, Figure 9(b)–(f)).

The comparison of photon count and signal-to-noise ratio (SNR) in the intermediate concentrations (Regime II) in simulations (*c* = 1.22 molecules/µm^2^) and in experiments (*c* = 10 pM) suggests that simulations where *t*_2_ = 100 ms have similar photon count and SNR as streptavidin-biotin experiments (see Figure S12).

### 4.3 Time-resolved measurements to benchmark against TIRF microscopy

**Figure 5:**
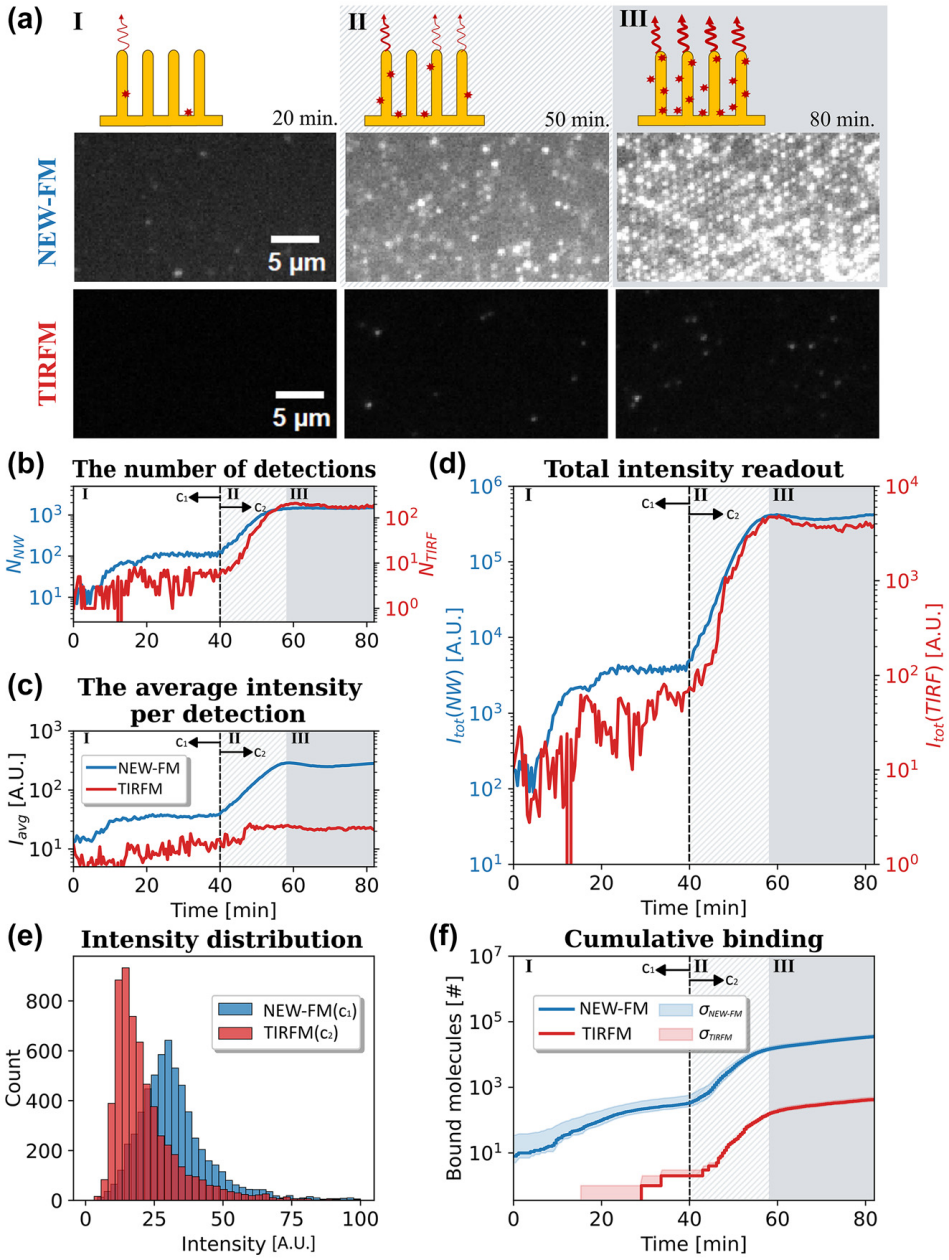
Comparison of NEW-FM and TIRFM. (a) Micrographs at different time points using NEW-FM on nanowires (top) and TIRFM on glass (bottom) demonstrating the three regimes of molecular binding (see main text). (b) The number of detections is depicted, with the count of bright nanowires represented in blue (*N*_NW_ on the left axis) and the detected molecules in TIRFM shown in red (*N*_TIRF_ on the right axis). (c) The average intensity *I*_avg_ in each frame. (d) The total intensity (*I*_tot_) in NEW-FM originating from nanowires (blue) and total intensity originating from molecules bound to planar glass in TIRFM (red). (e) The intensity distributions of detections. Note that for NEW-FM, this corresponds to *c*_1_ = 1 nM (Region I), and for TIRFM, it is *c*_2_ = 10 nM (Region III). (f) The cumulative binding (see Supplementary Methods, Eq. S(2) on how *N*_mol_ is obtained) as a quantification of bound fluorophores on nanowires (blue) and on glass (red).

One of the primary advantages of TIRFM is its capability to perform time-resolved single molecule detections of surface binding events, even in the presence of suspended fluorescently labeled molecules. Therefore, we conducted an experimental benchmark of time-dependent NEW-FM against TIRFM directly, in the very same microfluidic channel, enabling a direct comparison of StvA647 binding under identical experimental and imaging conditions (see Figure 1). The comparison centered on two distinct analyte concentrations, 1 nM and 10 nM, representing Regimes I and III, respectively, in Figure 5(a). It’s noteworthy to mention that while both concentrations fall within Regime III for titration experiments (see Figure 3(a)), variations in experimental conditions and channel designs can lead to differences in binding outcomes.

For the lower analyte concentration (Regime I, *t* < 40 min, *c*_1_ = 1 nM), we find that the number of detections on nanowires exceeds that on planar glass and monotonically increases until saturation is reached at around *t* = 20 min (Figure 5(b)–(d)). In contrast, the average intensity per detection does not change neither on nanowires nor on glass in this region (Figure 5(d)), indicating that only one molecule is bound at any identified location. Notably, the average intensity for TIRFM is more noisy and unstable than that of NEW-FM, reflecting that fewer binding events occur on glass in this regime.

At *t* = 40 min, a higher concentration of the analyte (*c*_2_ = 10 nM) is introduced, leading to a transition from a single-molecule binding regime (Regime I) to subsequent Regimes II and III on the nanowire substrate (see Figure 5(a)–(c)). The binding on glass is still resolved at the single-molecule level (Figure 5(b)–(d)). At around *t* = 60 min, no overall increase of intensity is observed on both substrates (Regime III in Figure 5(b)–(d)), suggesting that the surfaces have reached saturation. Across these two concentrations, TIRFM and NEW-FM display changes in the overall intensity spanning two and three orders of magnitude, respectively (Figure 5(d)). We estimate the cumulative binding rate on nanowires and glass (see Supplementary Methods, Section 1.6.2), which demonstrates that nanowire substrate is more sensitive to analyte capture at low coverage (Figure 5(b)–(d)), Region I, *c*_1_ = 1 nM), at which the overall number of bound molecules on glass is two order of magnitude lower. A significant response is observed on planar glass only in the higher concentration regimes, in which case multiple fluorophores are already bound to individual nanowires (Region II and III, Figure 5(a) and (c)).

Furthermore, a comparison of the intensity distribution of detections in the single molecule regimes (Regime I for NEW-FM and Regime III for TIRFM) reveals different patterns. NEW-FM displays a near normal distribution, whereas TIRFM appears to exhibit a truncated distribution, as depicted in Figure 5(e). There is also a twofold higher emission intensity at the peak of the distributions for NEW-FM compared to TIRFM. One can also conclude that the cumulative binding on glass and on nanowires in these single molecule regimes (see Supplementary Methods, Figure 12) resembles each other (Figure 5(f)).

## 5 Discussion

With the aim to make full use of the advantages of the “digital” character of vertically standing nanowires in NEW-FM, we introduced the single-nanowire localization pipeline NanoLoci and demonstrated its performance. This enabled the detection of concentrations ranging from 10 fM to 10 nM, representing a significant improvement of two to three folds compared to analyzing the same images without single-emitter localization techniques. In fact, the latter approach exhibit detection sensitivity only when a significant proportion of nanowires are bright. The detection improvement arising from signal digitalization is driven by two factors: single-emitter localization and frequency domain image analysis. Single-emitter localization starts with image filtering to reduce noise, making it easier to identify true positives. Next, single emitter candidates are determined through gradient thresholding, exploiting the higher local gradients of signals compared to noise, followed by PSF fitting to obtain subpixel resolution. Frequency domain techniques, relying on bright-field images and nanowire placement periodicity, improve the detection further by amplifying signals originating from nanowires while suppressing false detections from other regions of the substrate.

When benchmarking NanoLoci using simulated data with known ground truth, we find that our approach colocalizes the emerging signal with nanowire tips in high concentration regimes with relatively high accuracy. This accuracy in low concentration regimes is strongly dependent on the rate of photon emission, reaching 80 − 100 % if the number of emitted photons is sufficient. In our experiments, the estimated photon count per individual molecule is similar to the simulations with higher photon emission rate, suggesting reliable detection accuracy. However, achieving accurate localization becomes challenging at low SNR ratios at limited photon budgets [29].

Additional steps in NanoLoci, which utilize bright-field microscopy for image pre- and postprocessing, were able to effectively decrease the number of FPs and FNs, thus improving JI by 5 − 30 % in intermediate concentrations. However, additional challenges arise at intermediate concentrations, where half of the available nanowires have one molecule bound (1 molecule/µm^2^). In this case, the number of false negatives is significant, indicating that the deployed algorithms fail to accurately colocalize all bright nanowires. While no near-field interference effects should occur at 1 µm placement of nanowires, which could cause detection inaccuracies [11], the decrease in detection efficiency may be attributed to signal variation depending on the axial position of the bound molecule (see Figure S3(a) and (b)). Radially nonsymmetric signals may occur in instances of single molecule binding for the nanowire diameters and wavelengths used here, or when signals from neighboring nanowires overlap. These deviations can impact localization methods, which depend on symmetric Gaussian PSF fitting. Addressing these challenges necessitates exploration of sophisticated fitting algorithms capable of accommodating non-Gaussian PSFs, an application of image segmentation neural networks [42], [43], or choosing nanowires with different geometry and distribution. Image generation, overall, is a potent tool, serving not only to evaluate image analysis algorithm performance but can potentially facilitate efficient parameter optimization through machine learning, allowing to reduce user bias and enhancing analysis accuracy by systematically tuning parameters [44], [45], [46]. For this purpose, more realistic models for substrate-bound fluorophores and image background should be considered [11]. A fully rigorous study of the image formation and its dependence on the axial binding position of the fluorophore, including possible modification in the collection probability of emitted photons, is left for a future study.

The direct comparison of single StvA647 binding events using NEW-FM and TIRFM revealed a significantly higher binding rate on the nanowire (NW) substrate compared to planar glass. This difference may indicate shorter diffusion distances and thus shorter adsorption-induced concentration depletion distances in the liquid that flows between the nanowires [47]. Further, the respective prevalence of a normal and a truncated distribution for NEW-FM and TIRFM, respectively, indicates that a significant fraction of the binding events are below the background level when imaged in TIRFM. In contrast, NEW-FM seems to detect essentially all binding events occurring at the surface of the nanowires. A comparison of the signal distribution in single molecule regime also suggests an almost twofold higher sensitivity for NEW-FM compared with TIRFM, being attributed to a combination of excitation enhancement, lightguiding of the fluorescence emission, and directional emission from the nanowire tip [8], [16]. Further investigations across a wider range of concentrations and flow rates are necessary to fully comprehend this difference and these effects. In this context, it is worth noting that while benchmarking NEW-FM with TIRFM, the same objective with NA = 1.49 was employed. In fact, a smaller NA can lead to increased excitation enhancement for nanowires [14]. While image quality may be compromised using low-NA objectives, such an approach may allow the number of individually distinguishable nanowires in the FoV and imaging with less complicated microscope setups.

## 6 Conclusions

We conclude that there are four factors contributing to the impressively large dynamic range of NEW-FM. To begin with, the previously identified physical mechanisms that enhance the signal of fluorophores in the vicinity of waveguiding nanowires and that contribute to overall intensity increase, namely (i) the ability of nanowires to act as waveguides and to emit light directionally, (ii) excitation enhancement, and (iii) emission modification by quantum yield enhancement. However, as shown here, the major contribution to enabling high sensitivity and a large dynamic range is (iv) the unambiguous localization and the digital counting of nanowires at low concentrations, using an imaging pipeline such as NanoLoci. Specifically, we have shown that image analysis and single-emitter localization methods allow achieving detection sensitivity across a concentration range from 10 fM to 10 nM with two to three orders of magnitude increase in sensitivity when compared to analysis methods that only use intensity thresholding, but that do not use single-emitter localization. This highlights the significance of appropriate analysis techniques and bright-field imaging for nanowire localization in maximizing detection accuracy and streamlining both experimental and data analysis procedures.

This improved accuracy based on bright-field imaging and image fusion can also be applicable to fluorescence- or scattering-based imaging using other nanostructured surfaces or substrate-embedded nanoparticles, provided that the growth or placement pattern is periodic, as this allows for image pre- and postprocessing in the frequency domain. The image analysis software, NanoLoci, is designed with versatility in mind, enabling analysis of fluorescence images alone or incorporating image fusion with bright-field or darkfield data, making it adaptable to various nanostructured surfaces.

Nevertheless, challenges persist in accurately colocalizing signals with nanowire tips, particularly in noisy environments. Further investigation into the image formation of nanowire-fluorophore complexes is needed, focusing on understanding the influence of the axial binding position of the fluorophore and the focal plane of the microscope objective on colocalization accuracy. Larger lateral placements of nanowires can be simulated to explore the effect of nanowire distance in the optical detection of fluorescence signal. Polymer-embedded nanowires [8] or substrates with larger separation can allow overcoming the above-potential optical interference. Once appropriate lateral separation is ensured, the asymmetries in nanowire PSFs can open doors for three-dimensional molecular detection in NEW-FM [48], potentially allowing to observe 1D diffusion along nanowire *z*-axis using simple widefield microscopy.

The comparative direct benchmarking of NEW-FM against TIRFM revealed a notable twofold increase in sensitivity for NEW-FM in the single molecule regime, accompanied by a significant enhancement in the rate of molecular capturing on the nanowire substrate. We anticipate that these features, in conjunction with sophisticated image analysis pipelines compatible with ordered arrays of waveguiding nanowires of the type outlined in this work, may present unique opportunities for the future application of NEW-FM in bioanalytical sensing and medical diagnostics.

## Supplementary Material

Supplementary Material Details
