# Supplementary material for: Image analysis optimization for nanowire-based optical detection of molecules
Source: Nanophotonics. 2024 Sep 30;14(15):2563–74. doi: 10.1515/nanoph-2024-0243 (PMC12322720; doi:10.1515/nanoph-2024-0243)
Supplement: Supplementary file 1 — Supplementary Material Details [file j_nanoph-2024-0243_suppl_001.pdf]

Supplementary Material: Image analysis optimization for nanowire-based optical detection of molecules

R. Davtyan, et al

Contents

1 Supplementary Methods **2**
1.1 Sample preparation . . . 2
1.1.1 Experimental details for titration measurements . . . 2
1.1.2 Experimental details for time-resolved measurements . . . 3
1.2 Imaging simulations . . . 3
1.3 Image analysis pipeline for single molecule detection . . . 5
1.4 Image fusion based on frequency domain information . . . 6
1.5 Voronoi Tessellation . . . 8
1.6 Intensity evaluation metrics . . . 9
1.6.1 Titration measurements . . . 9
1.6.2 Time-resolved measurements . . . 9

2 Bright-field enhanced single nanowire detection with NanoLoci **10**
2.1 Threshold dependence of detection accuracy . . . 10
2.2 Detection accuracy across the simulated concentration range . . . 11

3 Supplementary Figures **13**
3.1 Image analysis pipeline . . . 13
3.2 Image generation pipeline based on the theoretical model . . . 14
3.3 The detected photon counts and signal-to-noise values of experimental and simulated images . . . 15
3.4 Time traces in single molecule regime . . . 16

4 Supplementary Tables **17**
4.1 Simulation parameters . . . 17
4.2 Simulated images . . . 17

1 Supplementary Methods

1.1 Sample preparation

Two different flow channels were used for single-frame titration and time-resolved measurements. Ibidi Sticky VI channel ($17 \times 3.88 \times 0.3$ mm^3) was utilized for titration measurements due to its simplicity, allowing multiple incubations at a same time in separate channels (see Fig. S1 (a-b)). However, due to the bulkiness of the nanowire substrate (fig. S1 (a)), laminar flow conditions cannot be achieved in this design. Thus, to ensure appropriate sample delivery and reliable imaging over long periods of time (see Fig. S1 (c-d)), we employed a custom channel where the thick substrate is incorporated into a poly(dimethylsiloxane) (PDMS) cavity.

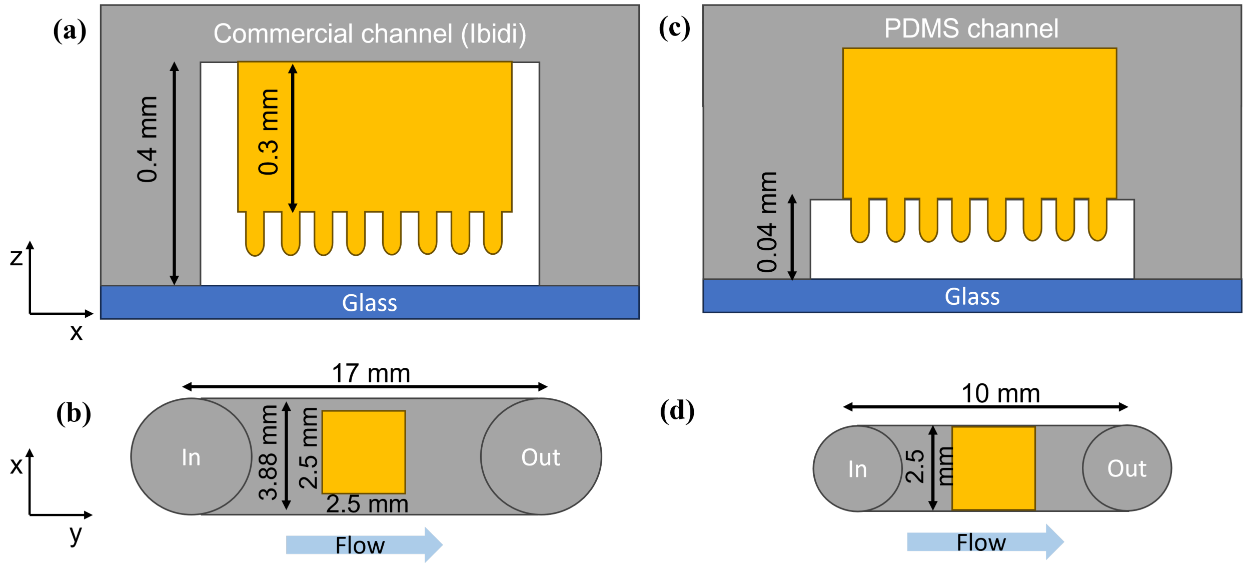

Fig. S1: (a-b) Commercial Ibidi sticky VI channel ($17 \times 3.88 \times 0.3$ mm^3) was used for titration measuruments. A nanowire device ($2.5 \times 2.5 \times 0.3$ mm^3) was attached inside the channel. (c-d) The custom PDMS channel used in time-resolved measurements.

1.1.1 Experimental details for titration measurements

GaP nanowire platforms of size $2.5 \times 2.5 \times 0.3$ mm^3 were attached to a 30 µL channel in a μ-Slide VI 0.4 microfluidic flow chamber (manufactured by ibidi, Germany) using silicone glue (Elastosil AO7, RTV-1 silicone rubber, Wacker Chemie AG, Germany). The assembly was then hermetically sealed with a microscope cover slide. After initial washes (140 µL per channel,$\times$ 3) with phosphate-buffered saline buffer (PBS) (pH 7.2, ThermoFisher Scientific, USA), a well-established streptavidin-biotin biorecognition assay was implemented. 120 µL of 6 µM biotinylated bovine serum albumin (bBSA, Sigma-Aldrich, USA) dissolved in PBS was added to each channel to functionalize the surface of the nanowire, since BSA strongly bonds to the coating SiO_2. bBSA was incubated for 1 hour, then rinsed with PBS (140 µL per channel,$\times$ 3). Reference images were taken at this stage (blank measurements).
Ten consecutive dilutions of AlexaFluor647 labeled streptavidin (StvA647, ThermoFisher Scientific, USA) were prepared from 1 mg/mL stock solution. Two nanowire devices were used for this experiment. Blank measurements were taken for each device individually, after the nanowire platforms were functionalized with bBSA and washed with PBS (see fig. S2). The functionalization step was performed once, and increasing concentrations of analyte (StvA647) were added: first, 0.01 pM and 0.1 pM concentrations of StvA647 were added to Device 1 and Device 2 respectively. After 1 hour of incubation at room temperature, the samples were washed (PBS, 140 µL per channel,$\times$ 3) and imaged. Afterwards, the incubation and washing steps were repeated for the following concentrations. Seven concentrations (0.01 pM, 0.1 pM, 1 pM, 10 pM, 1 nM, 10 nM, 100 nM) were prepared, but only the first six measurements were analyzed, as the microscope camera

pixels were saturated for the final 100 nM measurement.
For each concentration of StvA647, the detections were normalized on the appropriate blank measurement (Device 1 or Device 2).

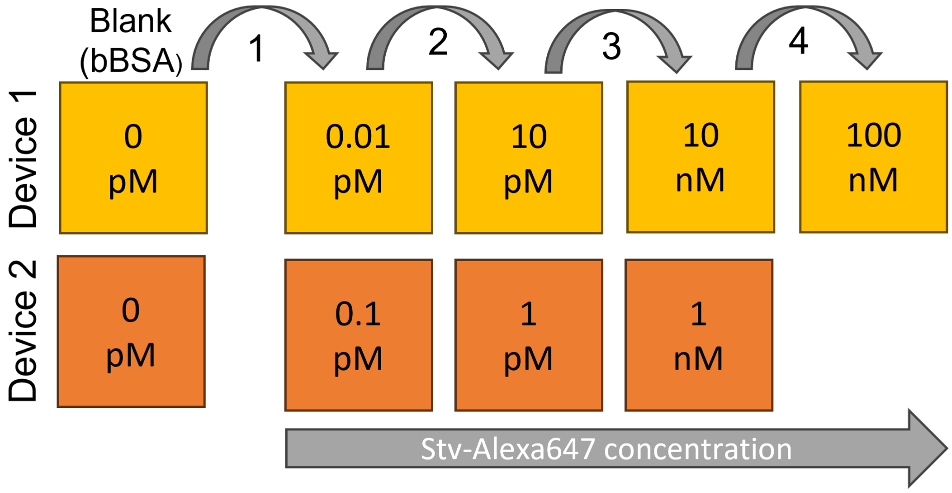

Fig. S2: Nanowire platform functionalization. Two nanowire devices in two different channels were used. The devices were functionalized with bBSA, then consecutive incubations and measurements with different concentrations of analyte were performed.

1.1.2 Experimental details for time-resolved measurements

For time-tesolved measurements, a custom device was fabricated using soft lithography. Poly (dimethylsiloxane) (PDMS) casting was manufactured by mixing the polymer and the curing agent (Sylgard 184 Silicone Elastomer Kit) in a ratio of 10: 1 on a custom-designed silicon template. The template with the mixture was then cured in an oven at 80° C for one hour, after which a PDMS device featuring a channel with dimensions of 10 mm $\times$ 2.5 mm $\times$ 40 µm. The device included a pocket of dimensions comparable to the nanowire substrate ($2.5 \times 2.5 \times 0.3$ mm^3). 3 mm inlet and outlet holes were created on top of the device. After treating the PDMS channel with air plasma for 10 seconds, and the glass slide for 50 seconds (Plasmatic Systems, Inc., North Brunswick, NJ, USA), the nanowire platform was attached inside the designated cavity using silicone glue (Elastosil AO7, RTV-1 silicone rubber, Wacker Chemie AG, Germany). The PDMS device containing nanowires and glass slide were sealed, then silicon tubes were attached as inlet and outlet reservoirs (see figure S2 (c, d)).
The same streptavidin-biotin assay was used for time-resolved measurements. After rinsing the device with PBS (300 µL, with 20 µL/min flow rate), a solution of 6 µM biotinylated bovine serum albumin (bBSA) dissolved in PBS was incubated using a single syringe infusion pump (Aladdin 1000, WPI) with a flow rate of 5 µL/min for 1 hour, followed by the washing step with PBS buffer for 30 minutes with a flow rate of 20 µL/min . 300 µL of 1 nM StvA647 was added to the channel and imaged over the course of 40 minutes. After 40 minutes, 300 µL of 10 nM StvA647 was added and imaged for another 40 minutes. Note that approximately 60 µL of 1 nM StvA647 was still present in the channel, when the highest concentration was added.

1.2 Imaging simulations

The signal enhancement in imaging mode depends on excitation enhancement (σ_{exc}) and quantum yield enhancement (σ_{QY}) by Purcell factor ($\text{C}_{\text{Purcell}}$) modification. σ_{exc}, describing the enhancement of the excitation intensity at the location of the fluorophore, depends on the wavelength of the incident light, incident angle, or in the case of microscope objective, the numerical aperture that controls the incidence angles used, as well as the position of the fluorophore [1]. See [2] for details on how σ_{exc} is calculated from modeling the electric field $|\vec{E}|^2$ at the fluorophore position.

The enhancement of quantum yield by Purcell factor can be defined as:

$$\sigma_{\text{QY}} = \frac{C_{\text{Purcell}}}{C_{\text{Purcell}} QY_0 + (1 - QY_0)},$$

where C_{Purcell} represents the modification of emission compared to a reference medium and is calculated by integrating the Poynting vector to obtain the power P_{em} emitted by the dipole:

$$C_{\text{Purcell}} = \frac{P_{\text{em},x} + P_{\text{em},y} + P_{\text{em},z}}{P_{\text{ref}}}.$$

Here, P_{ref} is the reference dipole power and is calculated analytically [2].
The optical image is obtained by near-field to far-field transformation (NFFT) using RETOP [3], with the assumption that the collection objective also functions as the imaging objective, enabling image formation in Fourier space (see fig. S3 (a)) [4]. After performing FFT, the electric field components (E_x, E_y, E_z) in far-field focused on the image plane are obtained. Under the assumption of polarization independence, $|E(x,y)_i|^2$, where the subscript i signifies the dipole moment orientation, represents the signal intensity of the optical image (note that we do not explicitly write the z-dependence of the binding position here). Denoted as $|E(x,y)|^2$, the sum of squared magnitude of electric field components represents the image intensity accounting for modifications in far-field emission intensity due to changes in dipole emission power, normalized by the total emitted power:

$$|E(x,y)|^2 = \frac{\sum_i |E(x,y)_i|^2}{\sum_i P_{em,i}}$$

$|E(x,y)|^2$ (see fig. S3 (b)) is then used as a probability distribution, from which individual photons are sampled [5]. Note, that in order to have an appropriate comparison with a free dipole, that is, a fluorophore in a homogeneous liquid surrounding, $|E(x,y)|^2$ should be normalized to that of a free dipole. However, in this case we did not model free fluorophores, but used a z-dependent Gaussian PSF as a model for substrate bound fluorophores [6, 7].
It can be observed in fig. S3 (b) that the shape of the intensity distribution in the modelled image is affected when the position of the fluorophore is varied on the z-axis. See [2] for the details and complete modeling pipeline.
σ_{exc} and σ_{QY} also vary depending on the axial position. The intensity of the image $|E(x,y)|^2$ is modified by multiplying it by σ_{exc} and σ_{QY} [2]. For simplicity, we assumed that the number of emitted photons (N) is modified by the same factors:

$$N_{enh} = N \cdot \sigma_{\text{exc}}(z) \cdot \sigma_{\text{QY}}(z)$$

For simplicity, we assumed that the probability to collect emitted photons does not depend on the axial binding position of the nanowire-bound fluorophore. Furthermore, we assumed that the probability to collect a photon from a nanowire-bound fluorophore is equal to the probability to collect a photon from a free fluorophore. The final image representing nanowire-bound fluorophores is constructed by summing the number of photons detected in each pixel, where the pixel size is determined by the microscope camera (fig. S3 (b)). The alteration of the numerical PSF, which refers to the intensity of the modelled image, may lead to either a sharply focused spot or a dispersion of photons around the nanowire.
When simulating a concentration of fluorophores, each fluorophore is randomly assigned to a substrate or a nanowire, on which a random z position is assigned (see fig. S10 for image generation workflow). The final image is constructed by adding the signal originating from nanowire and non-nanowire sources, such as substrate bound molecules, thermal noise, shot noise, etc. (fig. S3 (c)).

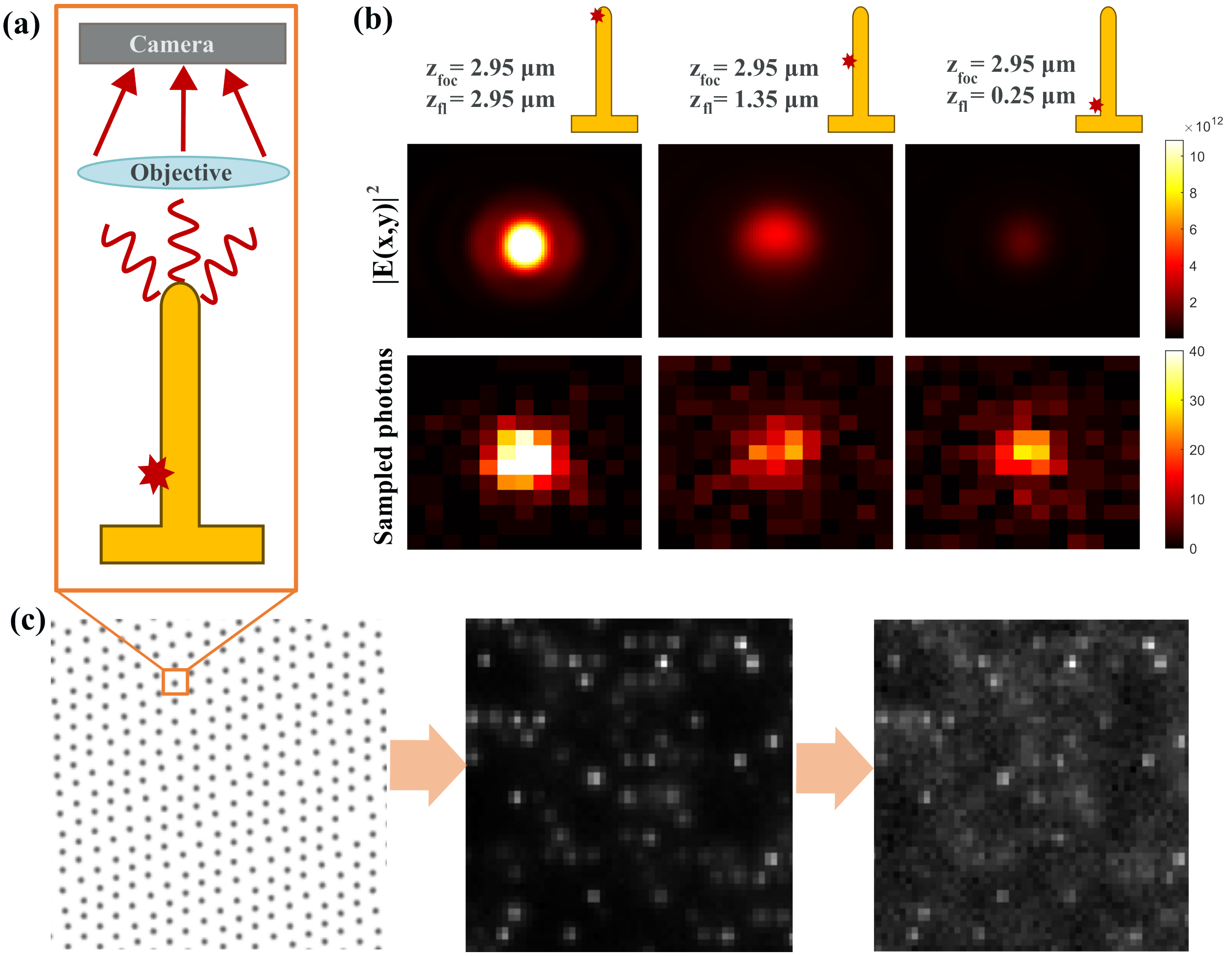

Fig. S3: (a) The optical image of a nanowire-bound fluorophore is obtained by NFFT and image creation in Fourier space. The sketch is adapted from [2], with the authors' permission. (b) Modeled image intensity $|E(x, y)|^2$ and the sampled photons, where the objective focal plane remains at the tip of the nanowires, but the position of a bound molecule is varied. The shape of numerical PSF, as well as σ_{exc} σ_{QY} depend on the axial position, but in this image only the variation due to the numerical change of PSF is demonstrated here. (c) The overall image is created by obtaining the optical image from each nanowire which has a fluorophore bound to it. The final image is the sum of the signal originating from nanowires and other sources in the system, such as surface bound molecules, and noise.

1.3 Image analysis pipeline for single molecule detection

As in traditional single-emitter localization methods, the image processing pipeline used here includes algorithms used to (i) identify bright spots which stand out from background according to certain mathematical criteria and (ii) estimate their coordinates in a given image frame [8, 9]. Here, we used a standard single-molecule detection pipeline image filtering (a-trous-wavelet, [10]) local-maxima estimation (local gradient, [11]), and position refinement (Gaussian PSF fitting, [12]).In addition to this analysis routine, in the case of standard biosensing experiments, where single-frame images are taken to determine molecular concentrations, we propose utilizing the geometrical properties of nanowire platforms by obtaining a bright-field image of a sample, serving as a ground truth for molecular binding positions without experimental complications. We suggest utilizing the periodicity of nanowires, by (i) enhancing the emitted signal from nanowire tips by using Fourier domain image fusion and (ii) excluding the signal not corresponding to nanowires by Voronoi triangulation.

Due to the assumed circular symmetry of the nanowire signal, the location estimation is based on local gradient calculation [11]. To identify individual nanowires, the local maxima are detected in the image by

finding the brightest pixels in each local neighborhood by image dilation [13]. The local gradient (G) is calculated for each detection according to [11, 14]:

$$G = \sum_{\text{box}} g_i \vec{u_i} \tag{S1}$$

where g_i is the central difference gradient at the i-th pixel, and $\vec{u_i}$ is the unit vector pointing toward the center of detection. The local gradient threshold (G_{min}) and user defined box are the only parameters that were changed in our analysis (see Figure S4b)).

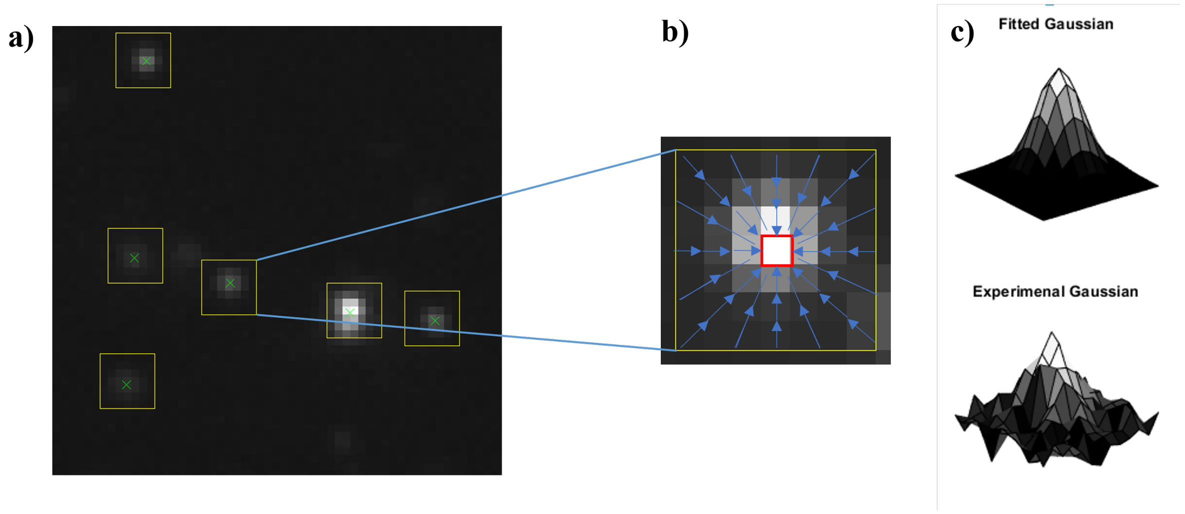

Fig. S4: a) A sample image with detected spots (yellow squares) and refined locations (green crosses). b) A demonstration of identifying the brightest pixel (red) with gradient lines pointing towards it and c) A sample Gaussian PSF function alongside with a noisy Gaussian PSF, which is descriptive of experimental signal.

Location estimation is followed by one of the most common techniques in single-molecule localization is still the fitting of 2D Gaussian (fig. S4c)) [12], which is incorporated in our analysis pipeline based on [15]. The location information obtained from fitting the Gaussian function is later used to calculate the average intensities of nanowires, followed by the calculation of total intensity as described in the main text.

1.4 Image fusion based on frequency domain information

Notch filtering, which is a common choice in digital image analysis to remove periodic noise from images or applied in spectroscopy for molecular or crystal structure analysis [16–18], is used to enhance the signal from fluorophores attached to nanowires in wide-field fluorescence images.

Let us denote fluorescence image as $A(x, y)$ and bright-field image as $B(x, y)$, denoting the coordinates in lateral space as (x, y) and coordinates in frequency space as (u, v). As described in [19], Notch filtering in Fourier space can be written as:

$$G'(u, v) = G(u, v) \circ (1 - H(u, v)),$$

where $G(u, v) = \mathcal{F}[B(x, y)]$ is the Fourier transform of the bright-field image and $H(u, v)$ is the band-reject notch filter, obtained by localizing peaks in FFT magnitude $G(u, v)$(see fig. S5 a)).

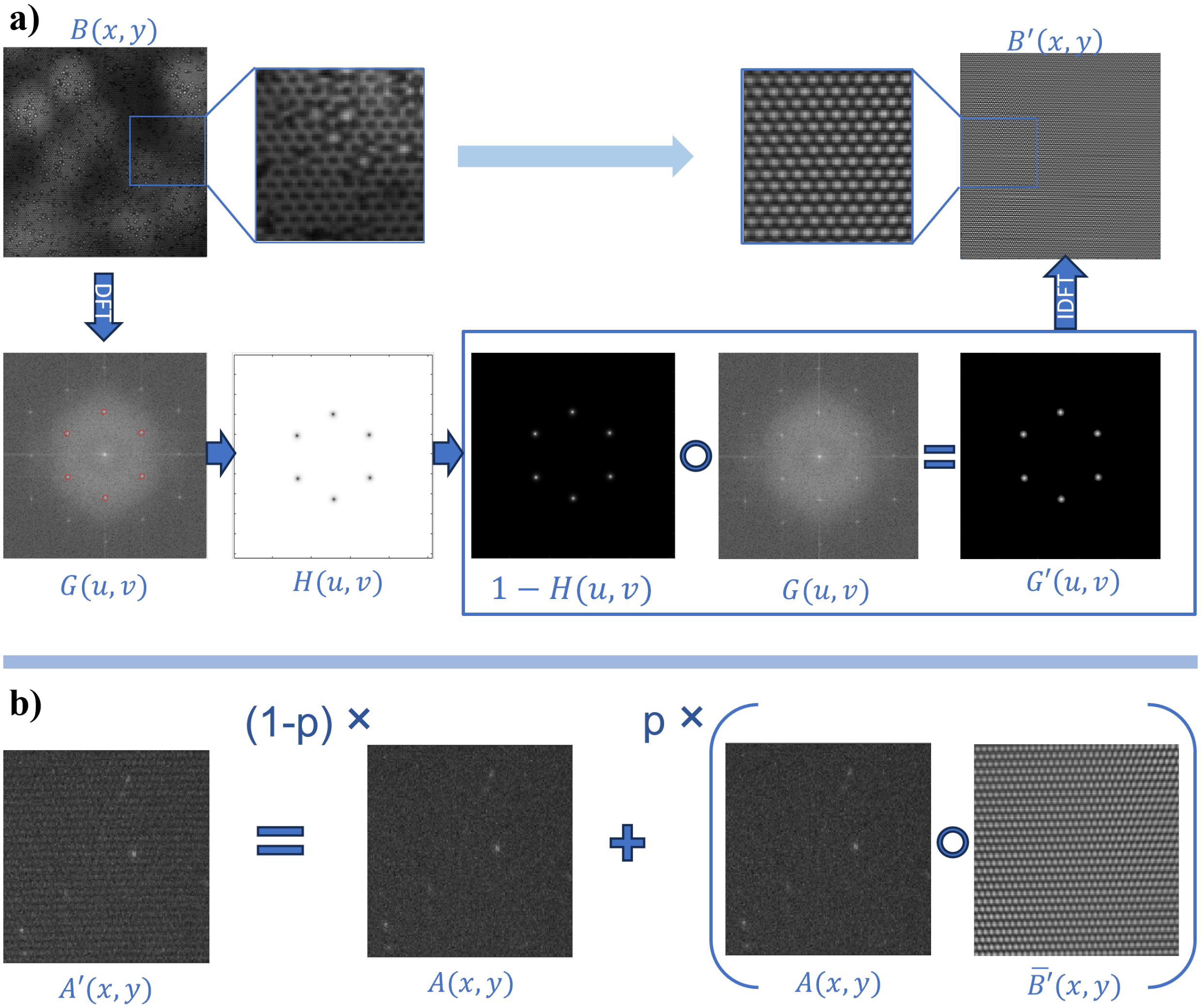

Fig. S5: **a)** Bright-field image $B(x,y)$ is transformed into Fourier domain and $G(u,v)$ is obtained. Frequency peaks are obtained from $G(u,v)$, from which the Notch band-reject filter $H(u,v)$ is created. The Hadamard product of the bandpass filter $(1 - H(u,v))$ and $G(u,v)$ results in a filtered frequency domain image $G'(u,v)$. After doing an inverse Fourier transform, $B'(x,y)$ is obtained, which represents the information of nanowire placement. **b)** The fused image $A'(x,y)$ is obtained by combining the raw image $A(x,y)$and the grid obtained in a). p is the percentage of the enhancement, and usually is selected in the range of [0.05, 0.1]

.

The filtered bright-field image $B'(x,y)$ is obtained from the inverse Fourier transform:

$$B'(x,y) = \mathcal{F}^{-1}[G'(u,v)]$$

The final image, $B'(x,y)$ contains only the information of hexagonal grid of nanowire placement and is then used to perform image fusion (see S5 b)):

$$A'(x,y) = (1-p) \cdot A(x,y) + p \cdot \left(A(x,y) \circ \bar{B}'(x,y)\right),$$

where $\bar{B}'(x,y) = \frac{B'(x,y) - \min(B')}{\max(B') - \min(B')}$ is $B'(x,y)$ rescaled to the range [0,1]. Here, $p \in [0,1]$ is the enhancement ratio, and is a user defined value. If $p = 0$, no enhancement is performed and the fused image $A'(x,y)$ is the same as the original image $A(x,y)$. After testing different values for p (not described here), we found that $p \in [0.05, 0.1]$, leads to the most accurate performance.

1.5 Voronoi Tessellation

We suggest considering the locations of nanowires as Voronoi seeds, and construct Voronoi cells [20–22] around them, where each cell will correspond to a local neighborhood of an individual nanowire (see fig. S6a)). Nanowire locations can be obtained from bright-field images, after performing band-pass Notch filtering according to fig S5. Note, that we assign Voronoi seed locations also to nanowires which are missing or kinked (seen as dark points in bright-field images) to ensure the correct partition of the plane into hexagonal regions.

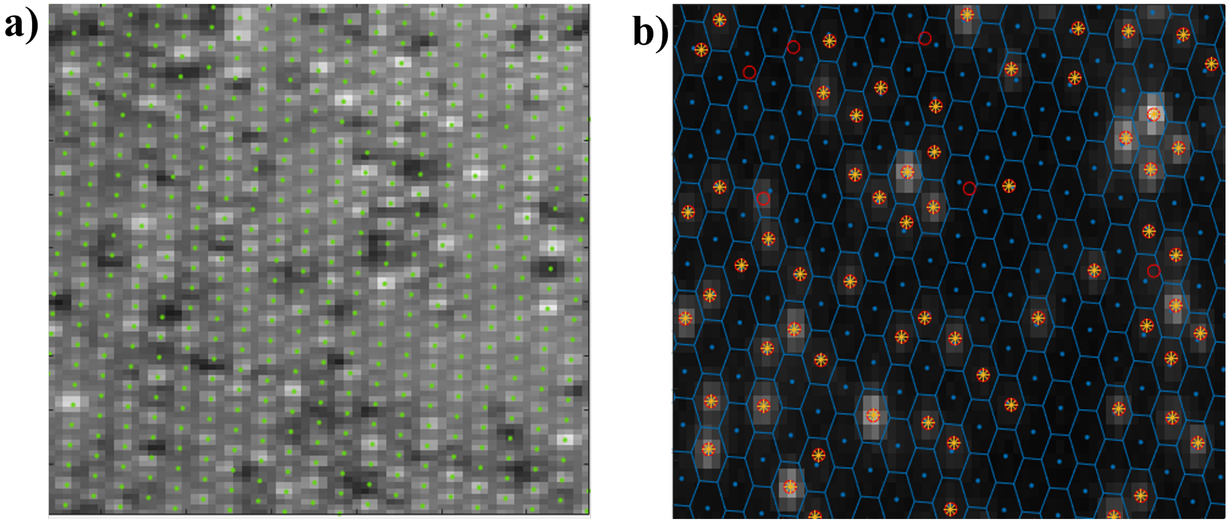

Fig. S6: A demonstration of Voronoi tessellation for mapping detections to nanowire locations. **a)** Shows a bright-field image, with nanowire locations marked in green. Note, that some missing nanowires (dark regions in the image) also have a corresponding nanowire center. This is to ensure the symmetry of Voronoi cells, and is based on the assumption of hexagonal growth mask. **b)** A Voronoi partition plane (blue grid) overlaid on a fluorescence image of nanowire-bound fluorophores. Unfilled red circles correspond to false positives: localizations which do not match to Voronoi seeds (blue dots). Orange asterisks are true positives: detections which are close to Voronoi seeds.

Algorithm 1 False-positive exclusion based on Voronoi tessellation

for each Voronoi cell **do**
  Calculate Center (x_c, y_c) of the Voronoi cell
  if $k_{fl} \neq 0$ **then**
    for each $(x_f, y_f) \in$ Voronoi cell **do**
      $D \leftarrow \sqrt{(x_f - x_c)^2 + (y_f - y_c)^2}$
      if D > threshold **then**
        remove (x_f, y_f)
      else
        keep (x_f, y_f)
      end if
    end for
  end if
end for

1.6 Intensity evaluation metrics

1.6.1 Titration measurements

For titration measurements, we compared the normalized intensities derived via NanoLoci using single-emitter analysis (as described in steps 1-3 in Fig. 2 , as detailed in the main text) with bulk pixel analysis methods.We considered two additional methods of analysis: total pixel intensity I_{px} and thresholded total pixel intensity I_{thr}. For an image of size $X \times Y$, the total pixel intensity was obtained using the formula:

$$I_{\mathrm{px}} = \sum_{i=1}^{X} \sum_{j=1}^{Y} (I_{ij} - I_{DC})$$

where I_{ij} is the intensity of each pixel, and I_{DC} is the microscope dark count. The other approach involves performing thresholding and calculating the sum of pixel intensities above the threshold [23]:

$$I_{\mathrm{thr}} = \sum_{i=1}^{X} \sum_{j=1}^{Y} \begin{cases} (I_{ij} - I_{DC}), & \text{if } (I_{ij} - I_{DC}) > \mathrm{thr} \\ 0, & \text{otherwise} \end{cases}$$

Here, the threshold is defined as:

$$\mathrm{thr} = \frac{1}{XY} \cdot I_{\mathrm{px}} + k \cdot \mathrm{std}(I - I_{DC}),$$

where the first term is the mean pixel intensity, $\mathrm{std}(I - I_{DC})$ is the standard deviation calculated for the entire range of intensity values and k is the thresholding parameter.
As in the case of single-emitter analysis, the normalization was done on the appropriate blank measurement, similar to eq. (3) in the main text:

$$I'_{\mathrm{px}} = I_{\mathrm{px}} - I_{0,\mathrm{px}},$$
$$I'_{\mathrm{thr}} = I_{\mathrm{thr}} - I_{0,\mathrm{thr}}$$

Note that the normalization is essential not only for estimating the signal level above the background, but also because of variations in the blank measurements across different nanowire platforms, as illustrated in the figure S2.
Naturally, normalizing the blank measurement against itself yields a result of 0. However, to accommodate the log-log scale of fig. 3 in the main text, we plot $N'_0 + 1$ and $I'_0 + 1$ as the blank measurement (dashed line) for ease of interpretation.

1.6.2 Time-resolved measurements

To facilitate direct comparison between the intensities of the detections obtained via NEW-FM and TIRFM, we subtracted the average intensity of the first frame separately from both measurements. This approach was used instead of subtracting I_{DC} from the obtained intensities, as seen in eq. (1) in the main text. This adjustment helps exclude the influence of the background signals, arising from measurements on two different surfaces—nanowire and glass.
In addition to single emitter localization in each time point as described in fig. S9, we estimate the number of newly bound molecules at a time $t+1$ on a nanowire i:

$$M^{+}(t+1, i) = \begin{cases} \left[\frac{\bar{I}_i(t+1) - \bar{I}_i(t)}{<I^{\mathrm{fl}}>} \right] & \text{if } \frac{\bar{I}_i(t+1) - \bar{I}_i(t)}{<I^{\mathrm{fl}}>} > 1 \\ 0 & \text{otherwise} \end{cases}$$

where [] indicates rounding up to the nearest integer value and $< I_{fl} >$ is the average-expected intensity of an individual molecule and is estimated from I_{avg}. It is obtained in Regime I, when only few nanowires are bright and the signal can be attributed to an individual molecule. From this estimate, also the approximate cumulative sum of surface bound molecules $N_{\mathrm{mol}}(t+1)$ for each consecutive time frame $t+1$ can be calculated:

$$N_{\mathrm{mol}}(t+1) = \sum_{t=1}^{t+1} \sum_{i=1}^{N} M^{+}(t+1, i) \tag{S2}$$

The number of molecules bound to glass in TIRFM is calculated in the same fashion.

2 Bright-field enhanced single nanowire detection with NanoLoci

In this section, we compare a sequence of conventional single-molecule algorithms with brightfield imaging enhanced nanowire detection, denoting the choice of algorithms as follows. Method 1 includes (i) image filtering using a-trous wavelet algorithm [10], (ii) local maxima estimation using image dilation and local gradient thresholding [11] and (iii) 2D Gaussian function fitting (see fig. S9 and fig 2, step 2). Method 2 includes the abovementioned steps, but additional Fourier domain image pre-processing is used (see figures S5, S9 and fig 1, steps 1-2). Method 3 extends Method 2 by including outlier exclusion via Voronoi tessellation (see fig. S6) to filter the detections which do not match the centers of nanowires (fig S9 and fig 2, steps 1-3). Method 3, which incorporates the complete pipeline of image analysis described here, is referred as *NanoLoci* later on.
The frequency domain analysis techniques are written in MATLAB and are freely available as a part of NanoLoci toolbox, alongside with the complete image analysis pipeline. The toolbox is available at `https://github.com/nanoRuby/NanoLoci`.

2.1 Threshold dependence of detection accuracy

To assess the efficacy of our methods, a simulated dataset is used to evaluate the accuracy of nanowire detection by comparing the detected locations with their known positions. This evaluation relies on varying two crucial parameters: the gradient threshold G_{min} and the matching distance between detections (see *Methods in the main text*). Once the fluorophores are localized in the fluorescence image (see Figure S6 a), these detections match the constructed Voronoi planes according to Algorithm 1. Due to the shift of optimal detection threshold to higher values (see figure S7), we always choose the threshold for Method 2 and Method 3 to be:

$$G'_{min} = (1 + p) * G_{min}, \tag{S3}$$

where G_{min} is the detection threshold of Method 1 and $p \in [0, 1]$ is the ratio of enhancement. We chose $p(t_1) = 0.05$ and $p(t_2) = 0.1$ and calculated JI, Precision and Recall for the whole simulated concentration range, where the lower and upper errors are defined by varying the matching distance threshold. Gradient threshold is systematically varied from 1 to 100, with each value initiating a localization process with Method 1, Method 2 and Method 3. For each gradient threshold G_{min}, the number of true positives (TPs), false positives (FPs), and false negatives (FNs) is estimated. Simultaneously, matching distances between d_m=1.2 ($\approx$ 0.21 µm) and d_m=1.5 ($\approx$ 0.26 µm) and pixels are explored, with a step size of 0.05 pixels.

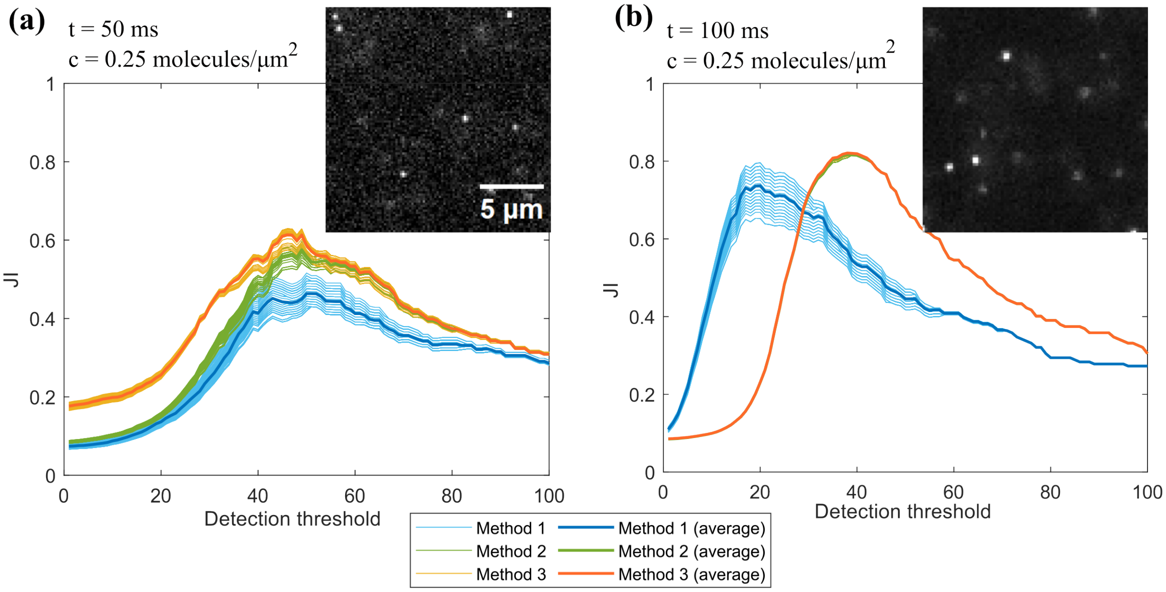

Fig. S7: Varying the gradient threshold (G_{min}) to test the robustness of analysis pipelines across the user-defined threshold range for (a) t = 50 ms and (b) t = 100 ms simulated exposure times. . Light blue, green and orange lines represent the variation of detection matching distance d_m in the range from 1.2 and 1.5 pixels for Method 1, Method 2 and Method 3, respectively. The darker blue lines represent the averaged JIs for all tested values d_m.

These parameter variations are conducted to assess the algorithm's robustness across different threshold settings. The Jaccard index (JI) serves as a metric to evaluate the accuracy of the detections. Notably, Method 2 employing image fusion in the Fourier domain consistently outperforms Method 1 resulting in a notable improvement in the Jaccard Index (JI) (see figs. S7). For the optimal detection thresholds ($G = [40, 60]$), a substantial 10% to 15% improvement in detection accuracy is achieved for $t = 50$ ms (see S7 (a)). Additionally, a further enhancement of approximately 5% was estimated when utilizing Method 3. While Method 3 did not demonstrate any improvement over Method 2 for higher photon emission rates (fig. S7 (b)), both of them still outperform Method 1 by 10%.
It is noteworthy that the peak in Jaccard Index (JI), indicating the threshold yielding maximum detection, is shifting towards higher thresholds with the implementation of image fusion, necessitating the use of higher thresholds for optimal performance. Moreover, the impact of varying d_m (distance matching threshold) was more pronounced in Method 1, leading to higher detection error rates. In Methods 2 and 3 this variance is largely reduced, as the deployment of image fusion reduces the dependence of detection accuracy on the chosen threshold (see fig S7). This tolerance to the choice of the detection distance threshold suggests that the detected locations are aligned better with the positions with nanowire tips.

2.2 Detection accuracy across the simulated concentration range

A comparison between detection accuracies for $t = 100$ ms and $t = 50$ ms across the whole concentration range was conducted, comparing Method 1 and Method 3 (NanoLoci). These values were computed for $d_m = 1.4$ px, with upper and lower errors calculated as $d_m^+ = 1.5$ px and $d_m^- = 1.3$ px, respectively (see fig. fig:JSC). As previously depicted in Fig. S7, Method (NanoLoci) significantly reduces the variation in detection accuracy attributed to changing d_m across all concentrations and intensities (see S8). While detections in high concentrations remain largely unaffected (c $>$ 1.22 molecules/µm^2 (see fig. S8)) when Method 3 is applied, the number of FPs and FNs is significantly decreased in intermediate concentrations (see fig. S8 (b-c), $0.05, 0.05, 0.25$ molecules/µm^2), leading to improved Precision and Recall (see fig. S8 (e-f)), which translates as an overall improvement in accuracy by $5-30\%$ depending on the simulated concentration and photon number (see fig. S8 (e-f)).

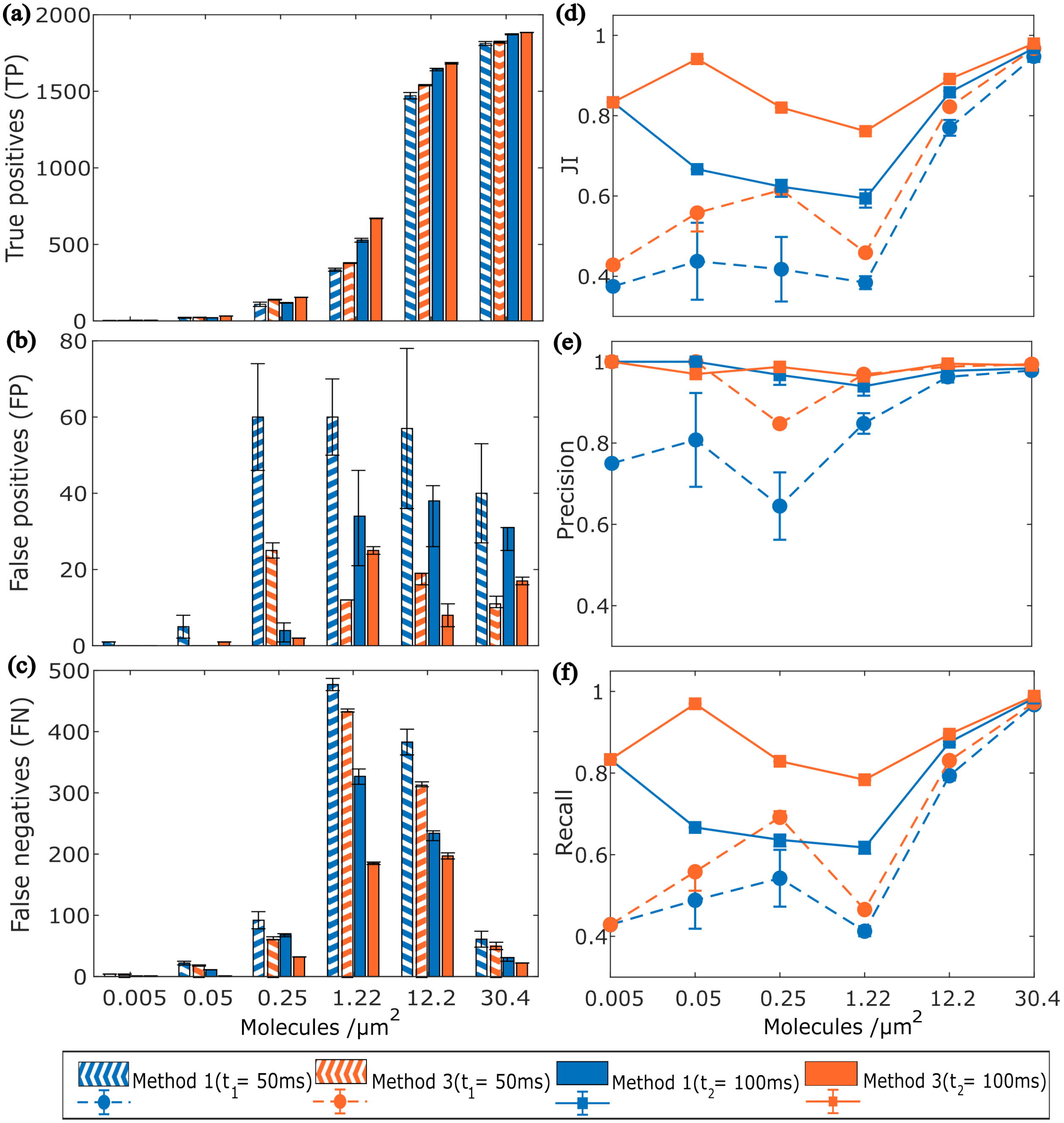

Fig. S8: A comparison of detection accuracies for $t = 100$ ms and $t = 50$ ms, comparing Method 1 (blue) and Method 3 (NanoLoci, orange). The values were calculated for $d_m = 1.4$ px and the upper and lower errors were calculated respectively for $d_m^+ = 1.5$ px and $d_m^- = 1.3$ px. (a) Both methods detect larger number of TPs, when the exposure time, thus the number of emitted photons, is higher. (b) The number of FPs is significantly higher for $t = 50$ ms with Method 1, meaning that there are detections which do not correspond to nanowire locations. With the application of Method 3, the number of FPs reduces for both conditions, leading to improved Precision (e). While for $t = 100$ ms the Precision is not affected significantly, the improvement is around 20% for $c < 1.22$ molecules/µm^2 for $t = 50$ ms. (c) Compared to Method 1, Method 3 reduces the number of FNs, especially in intermediate conditions, thus significantly improving the Recall (f) by $10 - 30$ % for concentrations c $= 0.005, 0.05, 0.25$ molecules/µm^2. (d) As Method 3 effectively lowers FP and FN rate while increasing the number of FPs, JI index is improved by around $5 - 30$ for concentrations c $= 0.05, 0.05, 0.25$ molecules/µm^2.

3 Supplementary Figures

3.1 Image analysis pipeline

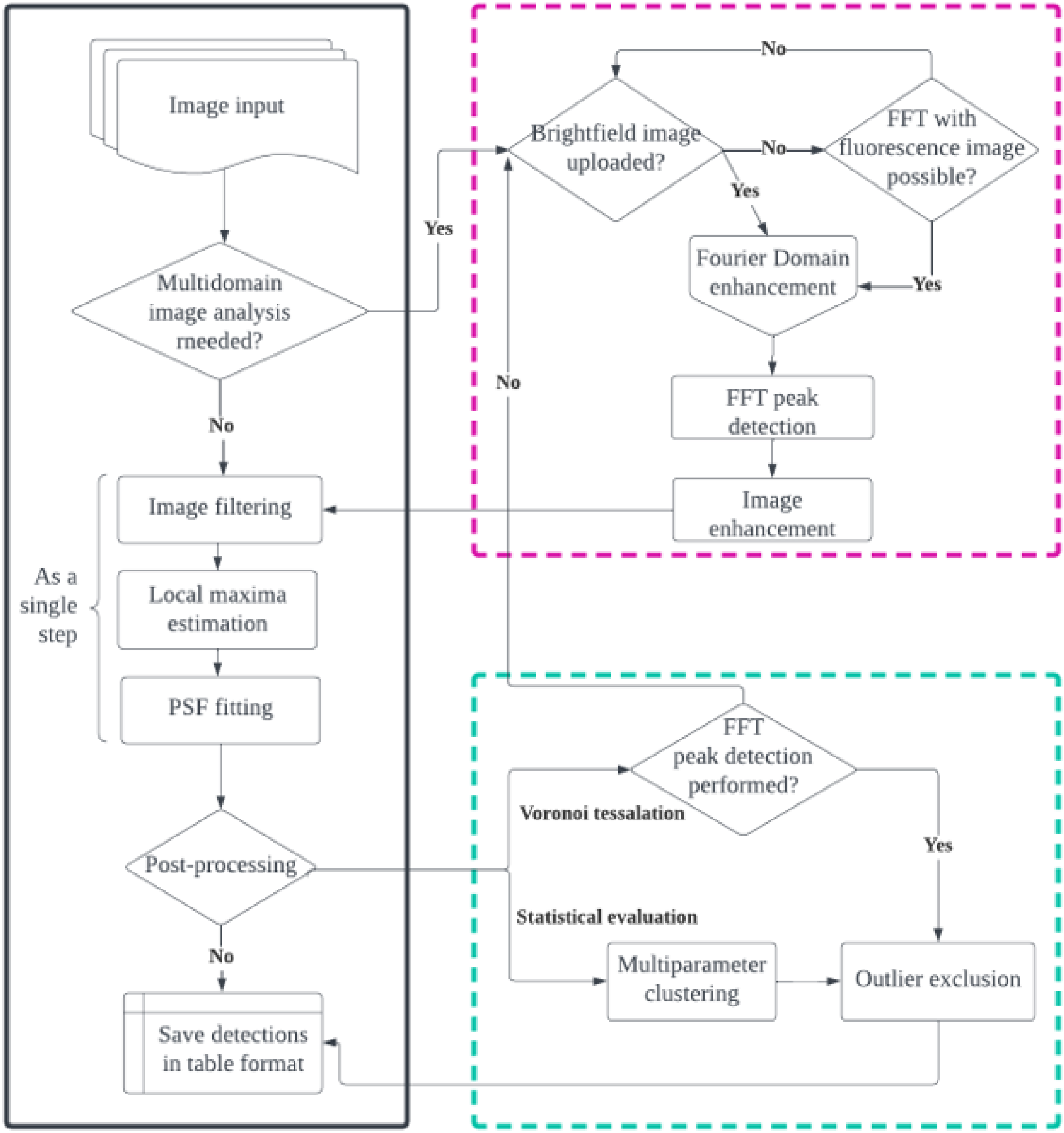

Fig. S9: The black box on the left corresponds to performing a conventional single spot detection using consecutive steps of image filtering, local maxima estimation and PSF fitting. The magenta and cyan boxes on the right correspond to additional image enhancement and outlier exclusion steps, which can be performed in the presence of bright-field image or if the number of bright nanowires in the fluorescence domain is high enough to identify peaks in the Fourier domain

3.2 Image generation pipeline based on the theoretical model

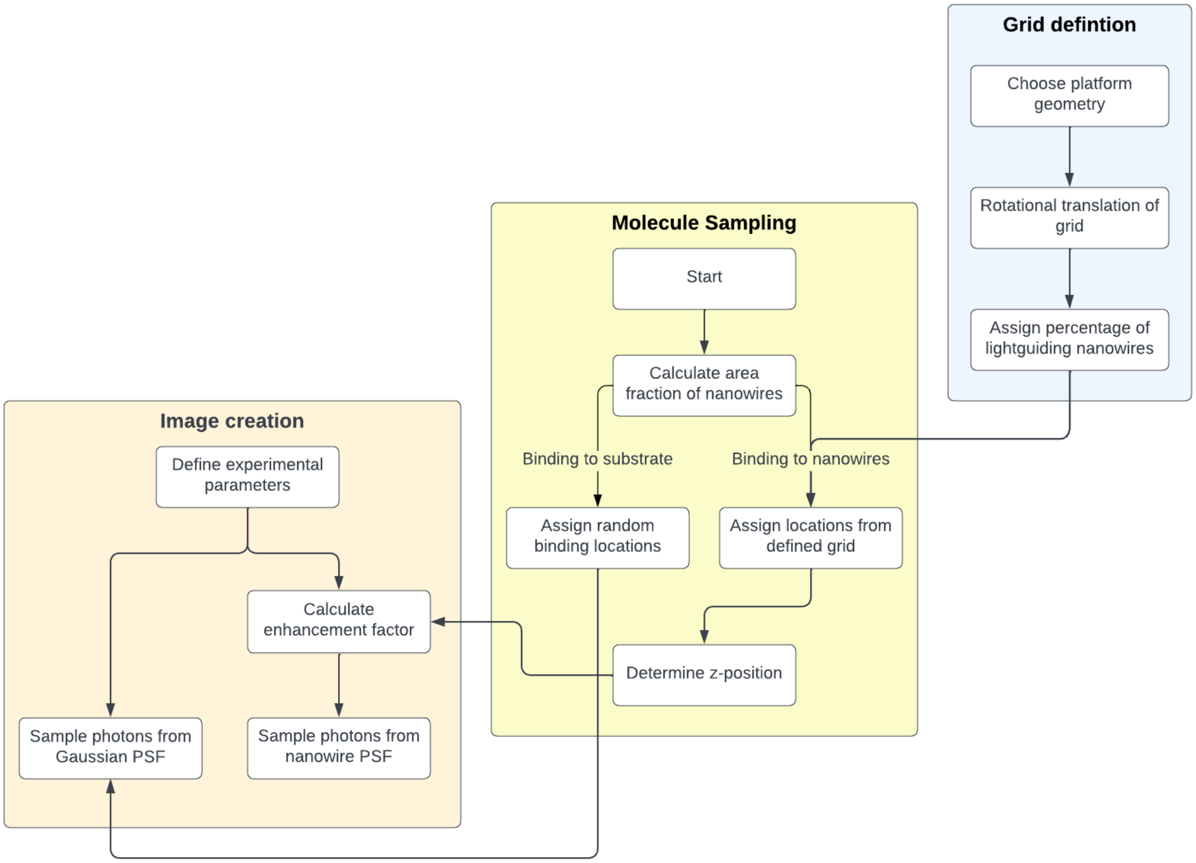

Fig. S10: The image simulation pipeline. 1. Grid definition: First, a grid corresponding to nanowire geometry is created, from which the ≈ 85% is chosen to be non-kinked and waveguiding as seen in table 1. This is done once per sample. 2. Molecule sampling: Independent of grid, the area ratio of nanowires and surface is calculated. Every time a new molecule is simulated, it is decided by area based random distribution where the molecule is bound. If it is bound to a nanowire, the z position is determined and 3. Image creation: given the enhancement factors at a given z, a number of nanowires is sampled from the appropriated PSF.

3.3 The detected photon counts and signal-to-noise values of experimental and simulated images

In order to compare the detected intensity levels in both titration experiments and simulations and to estimate which simulated imaging condition assembles the experimental dataset more, we compared the photon counts and signal-to-noise ratios (SNR) as can be seen in figure S11. We chose intermediate conditions (Regime II), to insure that the binding on nanowires is on the single molecule level on average, but enough detections are made to be able to compare photon count and SNR distributions.

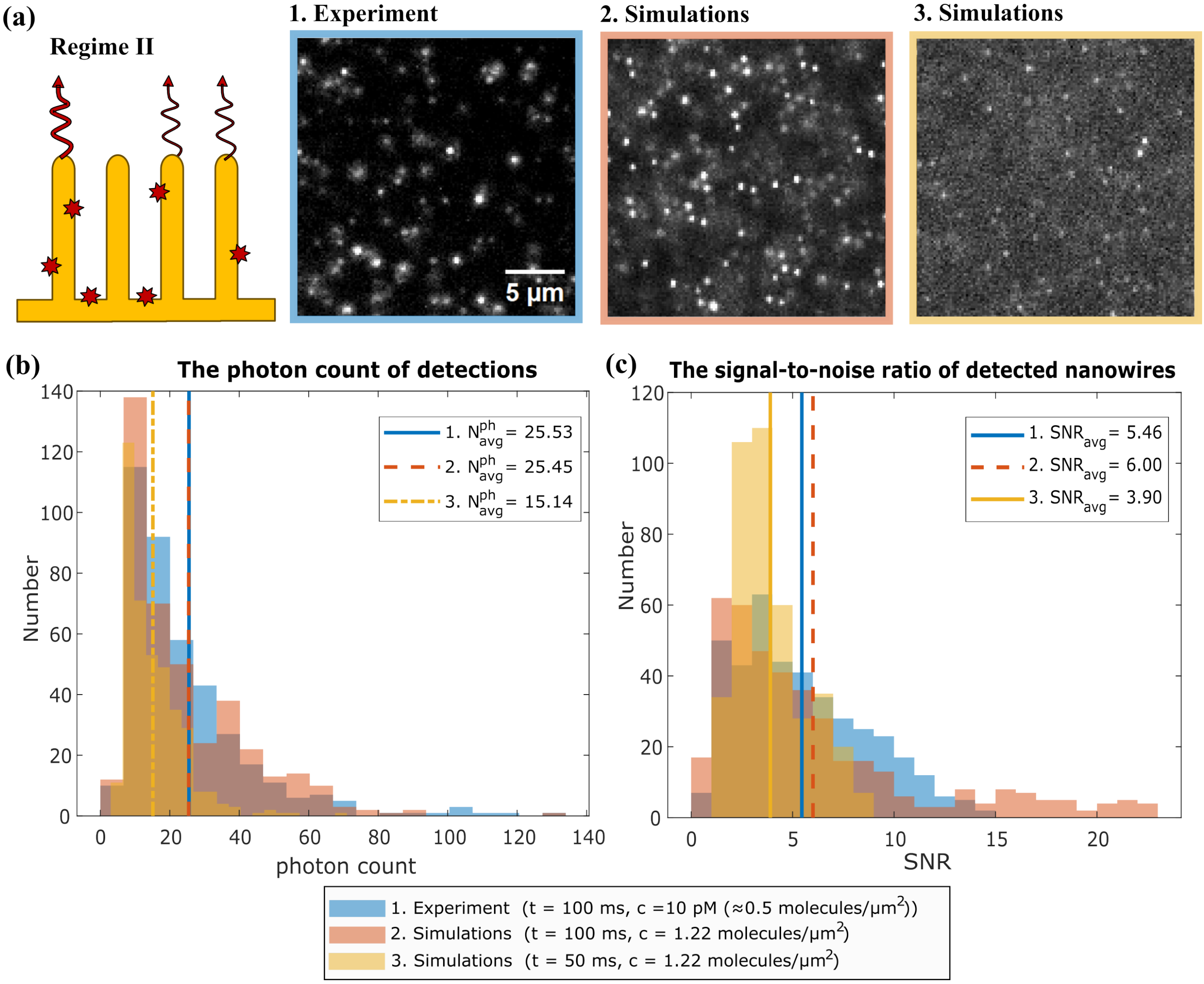

Fig. S11: In Regime II, where the large number of molecules are present, but a transition to multi-fluorophore binding did not occur yet, we compared (a) experimental images with simulations with t = 100 ms and t = 50 ms exposure time. From fitting a Gaussian PSF function according to [12, 15], (a) the number of detected photon in experiments and two simulation conditions is compared. When average photon number per detection (N^{ph}_{avg}) is compared, experimental conditions match well with t = 100 ms simulations conditions. The average number of detected photons is around 1.7 times less for t = 50 ms, which matches the simulated factor of 2. (c) When the signal-to-noise ratio of experiment and simulations is compared, experimental SNR_{avg} matches SNR_{avg}(t = 100 ms) better. However, although simulated nanowires have almost identical number of detected photons, they have larger SNR, which might indicate that experiments have higher intensity background.

3.4 Time traces in single molecule regime

This figure depicts time evolutions of randomly selected detections, where single molecule regime is observed. This can be seen from the step-wise bleaching behaviour, due to the fact that the analyte is labelled with three Alexa647 fluorophores on average.

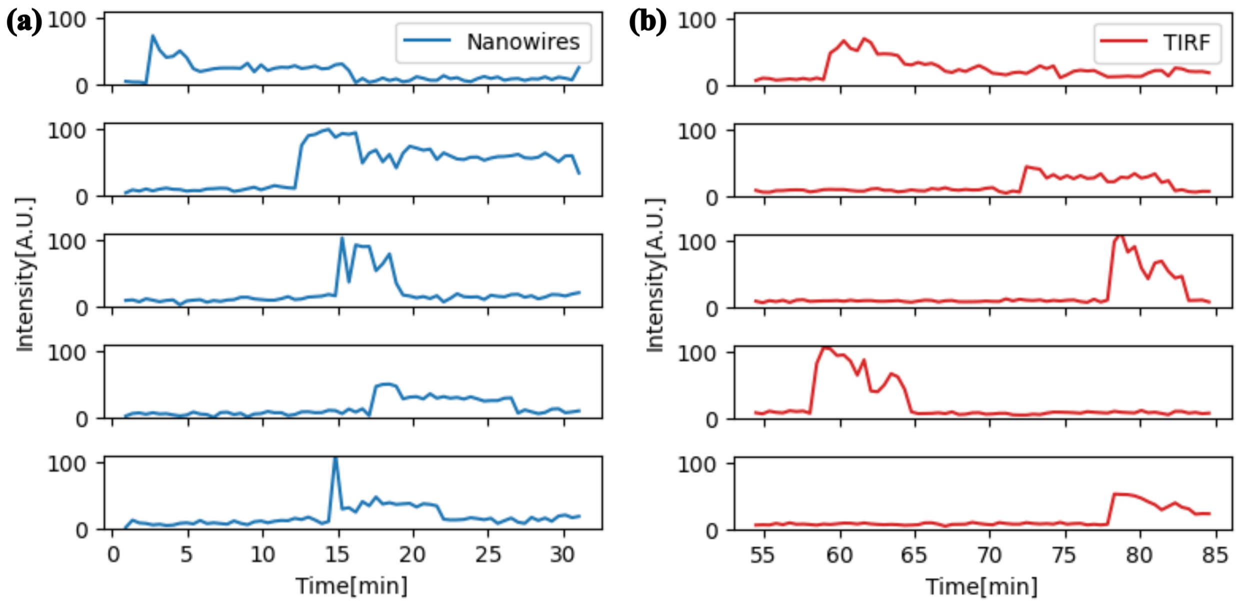

Fig. S12: Time traces of molecular binding on (a) nanowires in NEW-FM and (b) on glass in TIRFM. The analyte, StvA647, is labelled with three fluorophores on average, thus the bleaching can occur in multiple steps. Note, that the single molecule bleaching curves are obtained in different time points, as surface reactivity on nanowires and on glass varies, with accommodating more molecular binding.

4 Supplementary Tables

4.1 Simulation parameters

**FEM and Imaging simulation parameters**	
Diameter on the top, d_{top}, [nm]	100
Diameter on the bottom, d_{bot}, [nm]	100
Coating thickness, d_{coat}, [nm]	10
Length, L, [μm]	3
Fluorophore excitation wavelength, λ_{ex}, [nm]	640
Fluorophore emission wavelength, λ_{em}, [nm]	670
Fluorophore quantum yield, Φ	0.33
Objective numerical aperture, NA	1.2
Density of all nanowires, ρ, [#/µm^2]	1.19 ± 0.01
Density of straight nanowires, ρ_S, [#/µm^2]	0.99 ± 0.01
Nanowire spacing, p, [µm]	1

Table S1: The set of parameters used in finite element method (FEM) and image generation simulations.

4.2 Simulated images

	Surface concentration [molecules/µm^2]	The number of molecules on nanowires [#]	The number of molecules on the substrate [#]	The ratio of bright nanowires [%]	The approximate number of bright nanowires [#]
1	0.005	5	5	0.25	5
2	0.05	50	50	2.5	50
3	0.25	250	250	12.5	250
4	1.22	1250	1250	47	940
5	12.2	12500	12500	100	2000
6	30.4	31250	31250	100	2000

Table S2: Simulated concentrations and the corresponding number of bound molecules to nanowires and the substrate. The exact generated number of molecules is a random number sampled from a Poisson distribution, where the given number is the expectation (λ). The percentage of bright nanowires does not scale linearly with the concentration, as for intermediate concentrations, more than one molecule can bind to a single wire before all nanowires become bright.

References

[1] Ivan N. Unksov, Nicklas Anttu, Damiano Verardo, Fredrik Höök, Christelle N. Prinz, and Heiner Linke. Fluorescence excitation enhancement by waveguiding nanowires. *Nanoscale Adv.*, 5:1760–1766, 2023. doi: 10.1039/D2NA00749E. URL `http://dx.doi.org/10.1039/D2NA00749E`.

[2] Nicklas Anttu. Fluorophore signal and detection enhancement in nanowire biosensors. 2024. doi: https://doi.org/10.48550/arXiv.2403.16537. URL `https://doi.org/10.48550/arXiv.2403.16537`.

[3] Jianji Yang; Jean-Paul Hugonin; Philippe Lalanne. Light-in-complex-nanostructures/retop: Version 8.1, April 2020. URL `https://doi.org/10.5281/zenodo.3747487`.

[4] Lukas Novotny and Bert Hecht. *Principles of Nano-Optics*. Cambridge University Press, 2006. doi: 10.1017/CBO9780511813535.

[5] Tristan Ursell. Generate random numbers from a 2d discrete distribution. `https://www.mathworks.com/matlabcentral/fileexchange/35797-generate-random-numbers-from-a-2d-discrete-distribution`, December 2023. MATLAB Central File Exchange.

[6] Hendrik Deschout, Kristiaan Neyts, and Kevin Braeckmans. The influence of movement on the localization precision of sub-resolution particles in fluorescence microscopy. *Journal of Biophotonics*, 5:97–109, 1 2012. ISSN 1864063X. doi: 10.1002/jbio.201100078.

[7] Martin Lindén, Vladimir Ćurić, Alexis Boucharin, David Fange, and Johan Elf. Simulated single molecule microscopy with smeagol. *Bioinformatics*, 32:2394–2395, 8 2016. ISSN 14602059. doi: 10.1093/bioinformatics/btw109.

[8] Nicolas Chenouard, Ihor Smal, Fabrice De Chaumont, Martin Maška, Ivo F. Sbalzarini, Yuanhao Gong, Janick Cardinale, Craig Carthel, Stefano Coraluppi, Mark Winter, Andrew R. Cohen, William J. Godinez, Karl Rohr, Yannis Kalaidzidis, Liang Liang, James Duncan, Hongying Shen, Yingke Xu, Klas E.G. Magnusson, Joakim Jaldén, Helen M. Blau, Perrine Paul-Gilloteaux, Philippe Roudot, Charles Kervrann, François Waharte, Jean Yves Tinevez, Spencer L. Shorte, Joost Willemse, Katherine Celler, Gilles P. Van Wezel, Han Wei Dan, Yuh Show Tsai, Carlos Ortiz De Solórzano, Jean Christophe Olivo-Marin, and Erik Meijering. Objective comparison of particle tracking methods. *Nature Methods*, 11: 281–289, 3 2014. ISSN 15487091. doi: 10.1038/nmeth.2808.

[9] Mickaël Lelek, Melina T. Gyparaki, Gerti Beliu, Florian Schueder, Juliette Griffié, Suliana Manley, Ralf Jungmann, Markus Sauer, Melike Lakadamyali, and Christophe Zimmer. Single-molecule localization microscopy. *Nature Reviews Methods Primers*, 1(39):39, June 3 2021. doi: 10.1038/s43586-021-00039-w.

[10] I. Izeddin, J. Boulanger, V. Racine, C.G. Specht, A. Kechkar, D. Nair, A. Triller, D. Choquet, M. Dahan, and J.B. Sibarita. Wavelet analysis for single molecule localization microscopy. *Opt. Express*, 20(3): 2081–2095, Jan 2012. doi: 10.1364/OE.20.002081. URL `https://opg.optica.org/oe/abstract.cfm?URI=oe-20-3-2081`.

[11] Joerg Schnitzbauer, Maximilian T. Strauss, Thomas Schlichthaerle, Florian Schueder, and Ralf Jungmann. Super-resolution microscopy with dna-paint. *Nature Protocols*, 12:1198–1228, 6 2017. ISSN 17502799. doi: 10.1038/nprot.2017.024.

[12] Cameron S Smith, Nandakumar Joseph, Bernd Rieger, and Keith A Lidke. Fast, single-molecule localization that achieves theoretically minimum uncertainty. *Nature Methods*, 7(5):373–375, 2010. doi: 10.1038/nmeth.1449. Epub 2010 Apr 4.

[13] Jean Serra. *Image Analysis and Mathematical Morphology*, volume 1. Academic Press, 1982.

[14] Julia Valderas-Gutiérrez, Rubina Davtyan, Sudhakar Sivakumar, Nicklas Anttu, Yuyu Li, Patrick Flatt, Jae Yen Shin, Christelle N. Prinz, Fredrik Höök, Thoas Fioretos, Martin H. Magnusson, and Heiner Linke. Enhanced optical biosensing by aerotaxy ga(as)p nanowire platforms suitable for scalable production. *ACS Applied Nano Materials*, 5:9063–9071, 7 2022. ISSN 25740970. doi: 10.1021/acsanm.2c01372.

[15] Simon Christoph Stein and Jan Thiart. Trackntrace: A simple and extendable open-source framework for developing single-molecule localization and tracking algorithms. *Scientific Reports 2016 6:1*, 6:1–7, 11 2016. ISSN 2045-2322. doi: 10.1038/srep37947. URL `https://www.nature.com/articles/srep37947`.

[16] Najmeh Alibabaie and Ali Mohammad Latif. Fuzzy notch filter for periodic and quasi-periodic noise reduction in digital images, 10 2019. URL `https://papers.ssrn.com/abstract=3474858`.

[17] D. Chakraborty, M. K. Tarafder, A. Banerjee, and S. R. Bhadra Chaudhuri. Gabor-based spectral domain automated notch-reject filter for quasi-periodic noise reduction from digital images. *Multimedia Tools and Applications*, 78:1757–1783, 1 2019. ISSN 15737721. doi: 10.1007/S11042-018-6194-Z/FIGURES/16. URL `https://link.springer.com/article/10.1007/s11042-018-6194-z`.

[18] Igor Aizenberg and Constantine Butakoff. A windowed gaussian notch filter for quasi-periodic noise removal. *Image Vis Comput*, 26:1347–1353, 10 2008. ISSN 02628856. doi: 10.1016/j.imavis.2007.08.011.

[19] Rafael C. Gonzalez and Richard E. (Richard Eugene) Woods. *Digital image processing*. ISBN 9780133356724.

[20] Qiang Du, Max Gunzburger, and Lili Ju. Advances in studies and applications of centroidal voronoi tessellations, 2010. ISSN 20797338.

[21] Julie Bernauer, Ranjit Prasad Bahadur, Francis Rodier, Joël Janin, and Anne Poupon. Dimovo: a voronoi tessellation-based method for discriminating crystallographic and biological protein–protein interactions. *Bioinformatics*, 24:652–658, 3 2008. ISSN 1367-4803. doi: 10.1093/BIOINFORMATICS/BTN022. URL `https://dx.doi.org/10.1093/bioinformatics/btn022`.

[22] Franz Aurenhammer. Voronoi diagramsa survey of a fundamental geometric data structure. *ACM Computing Surveys (CSUR)*, 23:345–405, 9 1991. ISSN 15577341. doi: 10.1145/116873.116880. URL `https://dl.acm.org/doi/10.1145/116873.116880`.

[23] Pete Bankhead. *Introduction to Bioimage Analysis*. URL `https://bioimagebook.github.io/chapters/2-processing/3-thresholding/thresholding.html`.
